# Simultaneous B and T cell acute lymphoblastic leukemias in zebrafish driven by transgenic MYC: implications for oncogenesis and lymphopoiesis

**DOI:** 10.1038/s41375-018-0226-6

**Published:** 2018-08-15

**Authors:** Chiara Borga, Gilseung Park, Clay Foster, Jessica Burroughs-Garcia, Matteo Marchesin, Rikin Shah, Ameera Hasan, Syed T. Ahmed, Silvia Bresolin, Lance Batchelor, Teresa Scordino, Rodney R. Miles, Geertruy te Kronnie, James L. Regens, J. Kimble Frazer

**Affiliations:** 1 Section of Pediatric Hematology-Oncology, Department of Pediatrics, University of Oklahoma Health Sciences Center, Oklahoma City, OK 73104 USA; 2 Department of Women’s and Children’s Health, University of Padua, Padua, 35128 Italy; 3 Department of Pathology, University of Oklahoma Health Sciences Center, Oklahoma City, OK 73104 USA; 4 Department of Pathology, University of Utah and ARUP Institute for Clinical & Experimental Pathology, Salt Lake City, UT 84108 USA; 5 Center for Intelligence and National Security, University of Oklahoma, Norman, OK 73019 USA

**Keywords:** Cancer models, Oncogenes, Acute lymphocytic leukaemia

## Abstract

Precursor-B cell acute lymphoblastic leukemia (pre-B ALL) is the most common pediatric cancer, but there are no useful zebrafish pre-B ALL models. We describe the first highly- penetrant zebrafish pre-B ALL, driven by human *MYC*. Leukemias express B lymphoblast-specific genes and are distinct from T cell ALL (T-ALL)—which these fish also develop. Zebrafish pre-B ALL shares in vivo features and expression profiles with human pre-B ALL, and these profiles differ from zebrafish T-ALL or normal B and T cells. These animals also exhibit aberrant lymphocyte development. As the only robust zebrafish pre-B ALL model and only example where T-ALL also develops, this model can reveal differences between *MYC*-driven pre-B vs. T-ALL and be exploited to discover novel pre-B ALL therapies.

## Introduction

Acute lymphocytic leukemia (ALL), a common cancer and the most prevalent childhood malignancy, comprises >25% of pediatric neoplasia in the U.S., with ~85% being pre-B ALL [1, 2]. Relapses are all-too-common, making ALL the highest cause of pediatric cancer-related death [3]. Thus, there is a dire need for animal models of pre-B ALL to identify new molecular targets and discover new therapies, but efforts are impeded by a lack of in vivo models amenable to genetic and drug screens.

Zebrafish (*Danio rerio*) provide one potential solution, since they can model human leukemias accurately [4], have practical advantages (genetic tractability, high-throughput screens, cost), and share hematopoietic, oncogenic, and tumor suppressive pathways with humans [5]. These features permitted the creation of several zebrafish T cell ALL (T-ALL) models that mimic the human disease [6–10], which subsequently led to key findings in T-ALL genetics, disease progression mechanisms, and signaling [11–16], as well as facilitating screens for new treatment agents [17, 18].

However, despite the even greater clinical impact of pre-B ALL, effective zebrafish models lag behind. A single report of zebrafish B-ALL using transgenic *ETV6-RUNX1* [19] had low penetrance and long latency (~3% by 1 year), and no subsequent reports with this or any other B-ALL model exist.

Here we utilized a cell-specific fluorophore, *lck*:*eGFP* [20], that labels zebrafish B and T cells differentially to discover the first robust *D. rerio* B-ALL model. Surprisingly, B-ALLs occur in an already-established T-ALL model driven by transgenic human *MYC* [10], and they are so prevalent that many animals actually have coincident B-ALL and T-ALL. An intensive investigation of this new model using several approaches revealed a number of important findings. First, *hMYC*-induced B-ALL are pre-B subtype, express immature B cell transcripts, and like human pre-B ALL, spread aggressively to lymphoid and non-lymphoid tissues. Second, pre-B ALL express low levels of *lck*, and thus are dimly-fluorescent in these animals, unlike the brightly-fluorescent T-ALL of this model. Low *LCK* expression is conserved in human pre-B ALL. Third, in addition to their differential *lck*:*eGFP* expression, we report a two-gene classifier that distinguishes pre-B from T-ALL in *hMYC* fish. Finally, expression profiles of zebrafish pre-B ALL, T-ALL, and normal B and T cells revealed abnormal lymphopoiesis that may underlie the molecular pathogenesis of *hMYC*-driven ALL. In summary, we report a novel and robust pre-B ALL model, the first in zebrafish. Besides its value for genetic and drug screens, to our knowledge, *hMYC* fish represent the only animal model that develops both pre-B and T-ALL, providing a unique tool to explore molecular mechanisms of both human ALL types in the same genetic context, or even the same animal.

## Materials and methods

Zebrafish care and microscopy, FACS and flow cytometry analysis, qRT-PCR, RNA-microarrays, H&E, IHC, and WB analysis used standard techniques (see supplementary for details). Microarrays Data deposited at NCBI GEO repository GSE109437 (https://www.ncbi.nlm.nih.gov/geo/query/acc.cgi?acc=GSE109437).

### RNAscope-ultrasensitive in situ hybridization (RNA-ISH)

RNAscope (Advanced Cell Diagnostics-ACD, Hayward, CA, USA) fluorescent-field ISH used to detect *hMYC*, *cd79b*, and *lat* mRNA in fish sections. Procedure performed using the Multiplex-Fluorescent-Detection-Kit-v2 (#323110), according to manufacturer instructions (https://acdbio.com/). RNAscope probes used to specifically detect *human MYC* (#311761-C2), *D. rerio cd79b* (#511481) and *lat* (#507681). Probe labels (PerkinElmer, Waltham, MA, USA) as follows: TSA-Plus-Cyanine-3 (#NEL744001KT) for *hMYC* (yellow fluorescence), TSA-Plus-Cyanine-5 (#NEL745001KT) for *cd79b* (red), and TSA-Fluorescein (#NEL701A001KT) for *lat* (green). Slides imaged and analyzed using an Operetta High-Content Imaging System (PerkinElmer) and Harmony 4.1 software.

### Nanostring nCounter gene expression profiling

GEPs of FACS-purified GFP^lo^ and GFP^hi^ cell populations were quantified using a 96-gene Custom CodeSet according to manufacturer instructions (Nanostring nCounter Technologies, Seattle, WA, USA). Genes quantified using an nCounter Digital Analyzer and analysed using nSolver v3.0 software. Background thresholds defined by counts from a no-RNA blank that were subtracted from each sample. Raw counts were normalized to spiked-in positive control probes and housekeeping genes (*β-actin*, *eef1a1l1,* and *gapdh*), as suggested by the manufacturer. nSolver *t*-tests used to compare groups and identify differentially-expressed genes (FDR ≤ 0.05).

## Results

### Human *MYC* induces two zebrafish ALL types with distinct expression signatures

Mammalian *Myc*/*MYC* transgenes driven by a *D. rerio rag2* promoter induce zebrafish T-ALL [6, 10]. To detect and monitor ALL progression, we built double-transgenic fish by crossing Tg(*rag2:hMYC*) to Tg(*lck:eGFP*) fish, where a zebrafish *lck* promoter controls GFP expression [20].

**Fig. 1:**
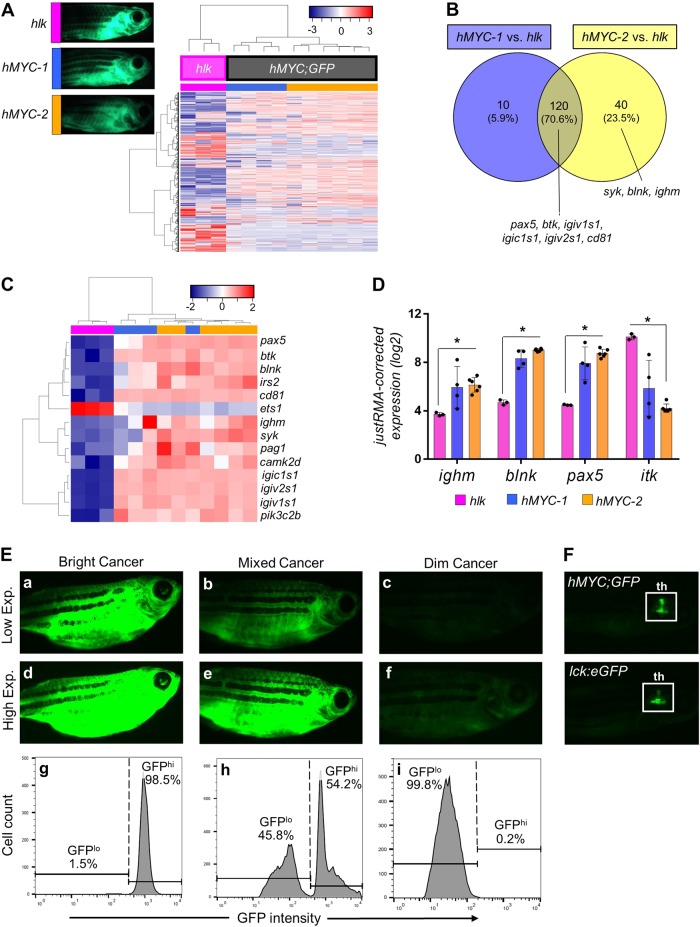
**Two ALL types in*hMYC* zebrafish with differing fluorescence intensities**. **A** Unsupervised analysis of 10 *hMYC* (grey) and 3 *hlk* (magenta) ALL, using highest-variance probes. *hMYC* ALL cluster into *hMYC-1* (blue) and *hMYC-2* (orange) groups. Representative fluorescent images of fish with ALL from each group shown at upper left. **B** Venn diagram of 170 over-expressed genes in *hMYC* ALL compared to *hlk* T-ALL. Genes up-regulated by both *hMYC-1* and −2 (*n* = 120) reside in the intersection, including six B cell-specific genes listed below the Venn diagram. Three other B cell-specific genes over-expressed by only *hMYC-2* ALL are listed below the yellow circle. **C** Unsupervised analysis using B cell-specific genes. **D** Expression of *ighm*, *blnk, pax5* and *itk* in *hlk*, *hMYC-1* and *hMYC-2* ALL. Each gene is significantly differentially expressed in *hlk* T-ALL vs. *hMYC-2* ALL (Mann–Whitney test, *p-value < 0.05). Expression values are log_2_ scale, normalized against the entire microarray dataset using the justRMA algorithm. Results shown as mean values ± standard deviation (S.D.). **E** Left: “bright” ALL, shown using low and high exposure settings (**a**, **d**). Cells are GFP^hi^ by flow cytometry (**g**). Right: “dim” ALL, using low and high (**c**, **f**) exposures. Cells are GFP^lo^ (**i**). Center: Fish with mixed-ALL (**b**, **e**), with discrete GFP^lo^ and GFP^hi^ populations (**h**). **F** Images of control *hMYC;GFP* (upper) and *lck:eGFP* (lower) fish with only thymic (th) fluorescence

Henceforth, we refer to this double-transgenic line as *hMYC;GFP*. The original report with *hMYC-*transgenic fish utilized tamoxifen to augment MYC activity via its fusion to a modified estrogen receptor;[10] in our study tamoxifen was not used. To study T-ALL in our system, we performed RNA microarray on FACS-purified GFP^+^ cells dissected from the bodies of 10 *hMYC;GFP* fish and 3 *hlk* fish [9], another zebrafish T-ALL model (see Fig. 1A for example animals).

Unsupervised analysis divided *hlk* and *hMYC;GFP* malignancies precisely, emphasizing fundamental differences in ALL from different *D. rerio* models (Fig. 1A). Unexpectedly, *hMYC;GFP* ALL also clustered into two subgroups with distinct gene expression profiles (GEPs).

To further investigate these groups, we used *hlk* T-ALL as a reference and designated the 4 ALL closest to *hlk* as *hMYC-1*, and the 6 ALL at the far right as *hMYC-2* (blue and orange samples in Fig. 1A).

Separate comparisons of *hMYC-1* or *hMYC-2* vs. *hlk* ALL revealed that B cell-specific genes were up-regulated by both types of *hMYC;GFP* ALL (*pax5, btk, cd81*, etc.; Fig. 1B), with *hMYC-2* ALL over-expressing additional B cell-specific genes (*syk, blnk, ighm*). Ingenuity Pathway Analysis™ (IPA) of these differentially-expressed genes showed enrichment and activation of “*PI3K-Signaling in B-Lymphocytes*”, “*B-cell receptor-signaling*”, and *“FcγRIIB-signaling in B-cells*” pathways by *hMYC-2* ALL, but not *hMYC-1*, relative to *hlk* T-ALL (data not shown). To further investigate the unanticipated expression of B cell genes by *hMYC* ALL, we repeated unsupervised analysis using only 14 statistically-significant B cell-specific genes. Remarkably, this signature classified *hlk* vs. *hMYC* ALL perfectly and largely reformed both the *hMYC-1* and *hMYC-2* subclasses (Fig. 1C).

Expression of B cell genes by *hMYC* cancers was unexpected, because B-ALL has never been described by several laboratories—including ours—that study transgenic *Myc*/*MYC* zebrafish [6, 10, 11, 15, 18, 21]. Yet microarrays clearly demonstrated B cell genes (*ighm*, *blnk*, *pax5*) were expressed at high, medium, and low levels by *hMYC-2, hMYC-1*, and *hlk* ALL, respectively, with T cell-specific *itk* showing the opposite pattern (Fig. 1D). We hypothesized *hMYC-1* and *hMYC-2* cancers might contain not only T-ALL cells, but also different fractions of B lymphocytes, accounting for these findings.

Specifically, we predicted that *hlk* cancers were “pure” T-ALL, whereas *hMYC-1* contained some B cells but mostly T-ALL cells, and that *hMYC-2* samples contained the highest percentage of B and/or B-ALL cells relative to T-ALL cells. Alternatively, leukemias can express aberrant markers [22], and *hMYC* might de-differentiate ALL, obscuring cell identities. In either case, B cell genes were highest in *hMYC-2* and detectable in *hMYC-1* also, so we next sought to definitively identify the cellular composition of *hMYC* cancers.

### B-ALL and T-ALL each occur in *hMYC;GFP* animals, with different GFP intensities

To definitively identify *hMYC;GFP* ALL as they first developed, we used serial fluorescent microscopy to monitor unaffected animals (i.e., fish lacking visible cancers). In young adults (3–6 months), we observed two phenotypes: brightly-fluorescent cancers originating in thymus and dimly-fluorescent cancers with variable thymic involvement (Fig. 1E, S1). To distinguish these, we used “low-exposure” settings that detected only bright cancers [Fig. 1E(a) vs. (c), S1A vs. S1B], and “high-exposure” settings that revealed dim ALL which were otherwise not visible [Fig. 1E(c) vs. (f), S1B vs. S1D]. Dim ALL differed from non-cancerous *hMYC* and *lck:eGFP* control animals that showed only normal thymic fluorescence (Fig. 1F).

**Fig. 2:**
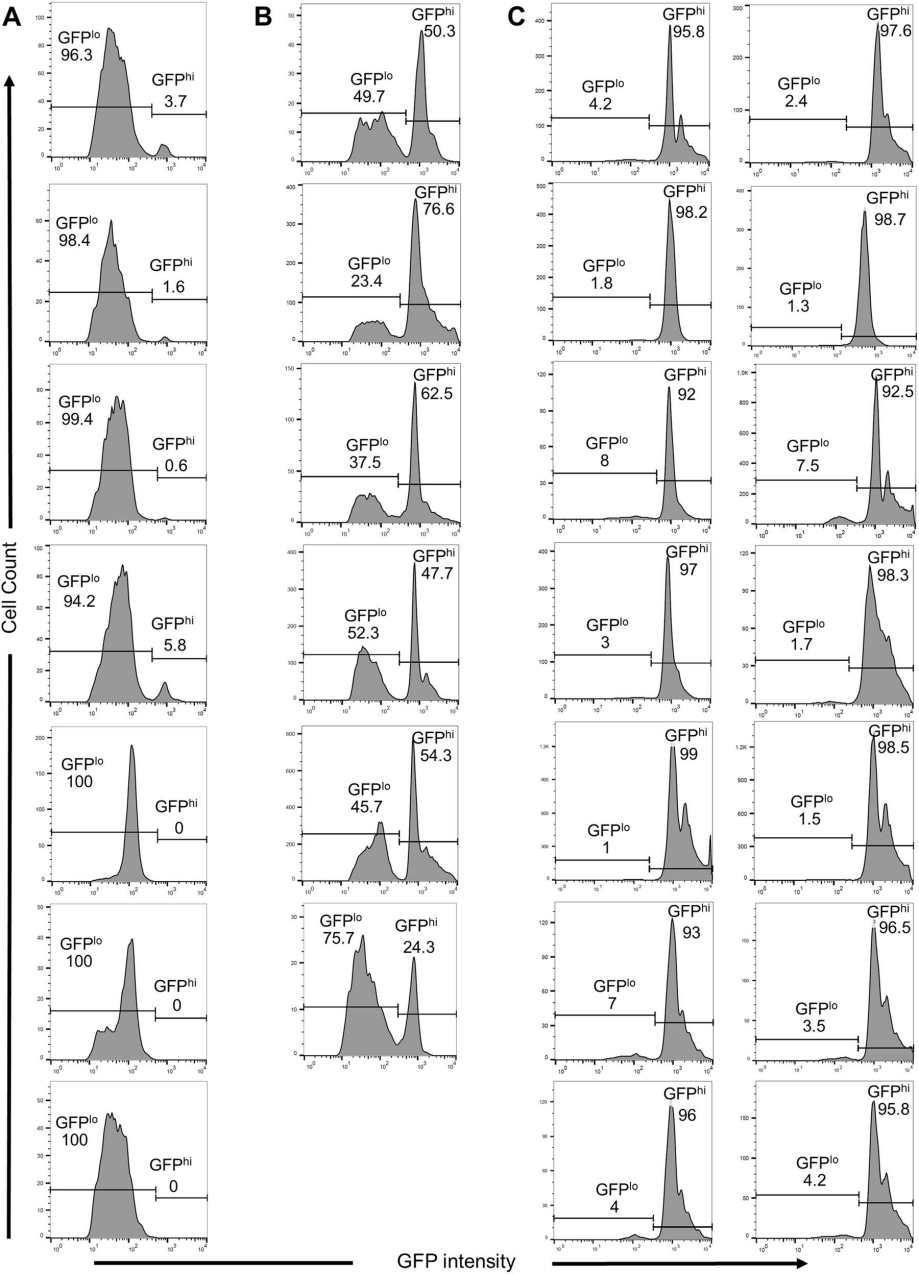
Distinct GFP intensities of *hMYC* dim and bright ALL. Flow cytometric plots of 33 ALL from 27 6-month *hMYC* fish: **A** 7 dim, GFP^lo^ ALL, **B** 6 mixed, GFP^lo^ & GFP^hi^ ALL, and **C** 14 bright, GFP^hi^ ALL

Microscopy findings were confirmed by dissecting GFP^+^ tissue from the bodies of these animals for flow cytometric analysis of GFP^+^ cells from the lymphocyte/precursor gate [23]. Bright and dim ALL showed distinct, >10-fold GFP intensity differences [Fig. 1E(g–i)]. Thus, we could discern ALL with only bright (>90% GFP^hi^ cells), only dim (>90% GFP^lo^ cells), or mixed cell populations (>10% for either minority GFP^hi^ or GFP^lo^ population). We analyzed ALL from 27 *hMYC* fish with fluorescent cancers at 6 months of age and found 7 dim ALL with near-exclusively GFP^lo^ cells (Fig. 2A) and 14 GFP^hi^-only ALL (Fig. 2C). Intriguingly, we also found 6 mixed-ALL that contained distinct populations of both GFP^hi^ and GFP^lo^ cells (Fig. 2B). Remarkably, these 27 animals developed 33 total ALL, 13 GFP^lo^ and 20 GFP^hi^. Of note, mixed ALL exhibited varying amounts of GFP^lo^ (23–76%) and GFP^hi^ (24–77%) populations; we attribute this to different onset dates and growth rates for each cancer. Thus, we predict these ratios change over time as each ALL independently progresses.

**Fig. 3:**
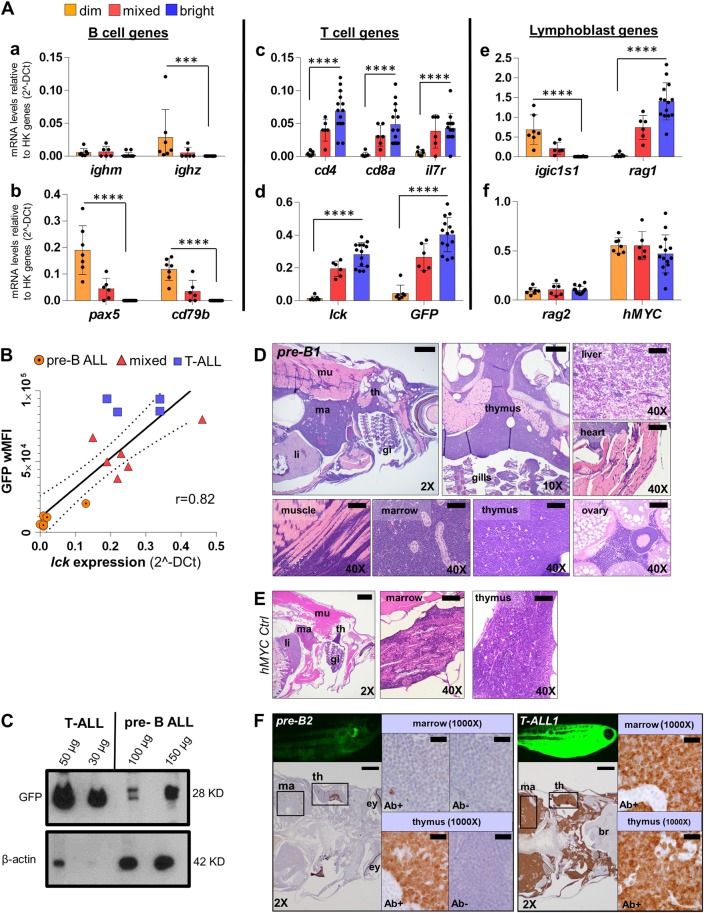
GFP fluorescence intensity of *hMYC* ALL correlates with B vs. T cell lineage. **A** qRT-PCR of ALL with differing GFP fluorescence (dim, *n* = 7; mixed, *n* = 6; bright, *n* = 14) of B cell-specific (*ighm*, *ighz*, *pax5*, *cd79b*; **a**–**b**), T cell-specific (*cd4*, *cd8a*, *il7r, lck*; c-d), lymphoblast- specific (*igic1s1, rag1, rag2*; **e**–**f**) genes and transgenes (*GFP*, d; *hMYC*, f). Results are normalized to housekeeping (HK) genes (*β-actin* and *eef1a1l1*) and shown as mean values ± S.D. Significant differences are indicated (Mann–Whitney test, *p-*values: ***<0.001, ****<0.0001). **B** Spearman correlation (*p*-value 0.0002, *r* = 0.82, *r*^2^ = 0.74) between *lck* vs. wMFI for: 7 B-ALL (circles), 6 mixed (triangles), and 4 T-ALL (squares). The solid line represents linear regression; dashed lines denote 95% confidence intervals. **C** Anti-GFP (#sc-9996) and anti-β-actin (#ab8227) WB of FACS-purified T- and pre-B ALL. **D** H&E of pre-B ALL infiltration in different sites and **E** *hMYC* control. **F** Anti-GFP (#GTX20290) IHC of *hMYC* pre-B ALL (*pre-B2*, left) and T-ALL (*T-ALL1*, right). 1000× images show staining with or without anti-GFP (Ab+; Ab-). th= thymus, ma= marrow, li= liver, mu= muscle, gi= gills, ey= eye, br= brain. 2× scale bar = 500 µm; 10× bar = 100 µm; 40× bar = 50 µm; 1000× bar = 20 µm

We next tested FACS-purified GFP^+^ dim, bright, or mixed-ALL cells for B cell- (*pax5*, *cd79b*, *ighz*, etc.), T cell- (*cd4*, *cd8*, *il7r*, etc.), and lymphoblast- (*rag2*, *igic1s1*, etc.) specific transcripts, as well as the *GFP* and *hMYC* transgenes by quantitative reverse-transcriptase PCR (qRT-PCR), analyzing all GFP^+^ cells as one population without separating GFP^lo^ and GFP^hi^ peaks. Dim/GFP^lo^ ALL expressed B, but not T, cell-specific genes [Fig. 3A(a–d), S2A(a–d)]. Low *lck* and *GFP* levels in dim ALL matched their weak in vivo fluorescence. Conversely, bright/GFP^hi^ cancers expressed only T cell genes. Mixed-ALL expressed both B and T cell genes at intermediate levels. Overall, expression correlated exactly with dim/GFP^lo^ vs. bright/GFP^hi^ phenotypes, and only mixed-ALL, which contained GFP^lo^ and GFP^hi^ cells, co-expressed genes of both cell types. Based on these findings, we conclude dim/GFP^lo^ ALL are B-lineage ALL. Mixed-ALL always exhibited distinct GFP^lo^ and GFP^hi^ cell populations (Fig. 2B) and expressed B-lineage and T-lineage genes [Fig. 3A(a–d), S2A(a–d)], so we deduce mixed-ALL are not biphenotypic, but simultaneous B-ALL and T-ALL in one animal.

B-lineage vs. T-lineage ALL could be unambiguously distinguished by *igic1s1* [Fig. 3A(e), S2A(e)], a predicted homologue of the *IGLL1* surrogate Ig light chain gene expressed by only immature B cells [24]. Only dim and mixed-ALL expressed *igic1s1*, but every ALL showed similar levels of the V(D)J recombination enzyme *rag2* [Fig. 3A(f), S2A(f)]. Based on *igic1s1* and *rag2* results, which only immature B cells co-express, we believe *hMYC* B-ALL are an immature B cell cancer, most likely pre-B ALL. A zebrafish *rag2* promoter regulates *hMYC*, so it is logical that *rag2*^*+*^ B-lymphoblasts (i.e., pre-B cells) are affected, just like T-ALL in this model [6, 10]. Moreover, similar *hMYC* levels in pre-B and T-ALL [Fig. 3A(f), S2A(f)] indicate this transgene has similar oncogenic potency in both lymphocyte lineages. Surprisingly, *rag1* was much lower in pre-B ALL [Fig. 3A(e), S2A(e)], making *igic1s1* and *rag1* a two-gene panel that can distinguish *hMYC* ALL types independent of *lck* or *GFP* levels. Mammalian B-lymphoblasts and T-lymphoblasts co-express RAG1 and RAG2, so dichotomous *rag1* levels were unexpected. However, unlike mammals, *rag2*-mutant zebrafish lack T cells, yet retain functional B cells [25]. How *D. rerio* B cells develop in this context is unknown, because if *rag1* is absent in B cells, only mutant Rag2 would be present to mediate immunoglobulin (Ig) recombination. Potential explanations for this paradox include compensatory *rag1* expression by B cells, expression of *rag1* at a different B cell stage, hypomorphic function of the truncated mutant Rag2 protein, or the formal possibility that zebrafish utilize a different V(D)J recombination mechanism than mammals.

As predicted by different in vivo fluorescence [Fig. 1E (a–f), S1] and GFP^lo^ vs. GFP^hi^ cytometric results [Fig. 1E(g–i), 2], *lck* and *GFP* also differed markedly between pre-B and T-ALL [Fig. 3A(d), S2A(d)]. A zebrafish *lck* promoter regulates GFP [20], and pre-B ALL expressed little *lck* or *GFP*, while T-ALL expressed both abundantly. In agreement, *lck* mRNA correlated with weighted median fluorescence intensity (wMFI; Fig. 3B), with each ALL type clustering separately, proving *lck* levels—and thus, cellular fluorescence—distinguish pre-B vs. T-ALL in this model. We also confirmed *GFP* mRNA and protein levels agree by Western blot (WB), with much higher amounts of total protein needed to detect GFP in pre-B ALL compared to T-ALL (Fig. 3C).

*lck* is generally considered to be T cell-specific [20], but zebrafish NK and myeloid cells also express *lck* [26, 27]. Pertinent to our study, we analyzed public data from different maturation stages of human lymphocytes [28] and found pre-B cells expressed higher *LCK* than naïve and mature B cells, although below that of T cells (Fig. S2B). Furthermore, Microarray Innovations in Leukemia (MILE) data [29] from human patients showed that although mean *LCK* levels are higher in T-ALL than pre-B ALL (Fig. S2C), 37% of T-ALL (64/174 patients) expressed equivalent *LCK* to 83% of pre-B ALL cases (409/493; red box in Fig. S2C), demonstrating *LCK* is not specific to T-ALL.

This is further exemplified by comparing the highest *LCK*-expressing pre-B ALL quartile to the lowest T-ALL quartile in this dataset, for which no statistical difference exists (*p-*value = 0.75; Fig. S2D). Thus, human and zebrafish pre-B ALL both express *LCK*/*lck*.

### Zebrafish pre-B ALL resembles human pre-B ALL morphologically

To examine pre-B ALL histology, we analyzed *hMYC* fish with dim ALL. Hematoxylin and eosin (H&E) stains showed lymphoblast infiltration of the kidney-marrow, thymus, liver, and elsewhere (Fig. 3D, S3A). Marrow hypertrophy was often profound, with marrow expansion and invasion through muscle into subcutaneous tissue and skin (Fig. S3A, *pre-B3*, *-B4*). Yet despite these massive disease burdens, fish remained only dimly fluorescent. As in humans, pre-B and T-ALL were indistinguishable by H&E (Fig. S3A–B), and both were markedly abnormal compared to control animals (Fig. 3E, S3C).

Immunohistochemical analysis (IHC), however, could discriminate pre-B from T-ALL, with very faint anti-GFP staining in GFP^lo^ ALL vs. strong signals in GFP^hi^ ALL (Fig. 3F, *pre-B2* vs. *T-ALL1*, S4A-B), and only remnant thymic tissue showing strong signals in pre-B ALL fish (Fig. 3F, *pre-B2* and S4A, *pre-B5, -B6*). Consistent with this, regions stained weakly by anti-GFP (Ab+) corresponded to dimly-fluorescent anatomic regions (Fig. 3F, S4A; 1000X panels).

**Fig. 4:**
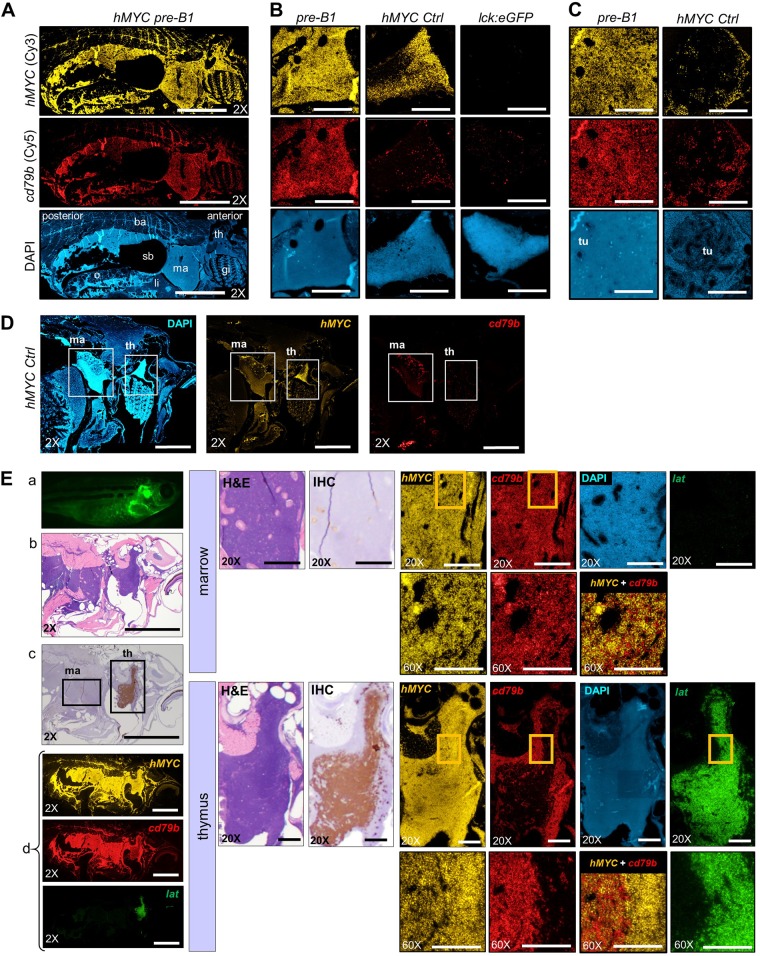
Pre-B ALL co-express *hMYC* and B cell-specific *cd79b*. RNA ISH for *hMYC* (yellow) and *cd79b* (red) in: **A** sagittally-sectioned *hMYC* pre-B ALL (*pre-B1*; scale bar = 2 mm), **B** Thymi of *pre-B1* (left), *hMYC* control (center), and *lck:GFP* (WT) control (right; scale bar = 200 μm), **C** Kidney-marrow of *pre-B1* (left) and *hMYC* control (right). Kidney tubules (tu) are displaced by *pre-B1* ALL cells in marrow DAPI image (scale bar = 200 μm), **D** *hMYC* control (scale bar = 1 mm). **E** Second *hMYC* fish with pre-B ALL and localized thymic T-ALL. Left**:** (**a**) High-exposure microscopy, (**b**) H&E, (**c**) anti-GFP IHC, (**d**) RNA ISH for *hMYC* (yellow), *cd79b* (red) and *lat* (green). Middle**:** high-power of kidney-marrow (top) and thymus (bottom) by H&E, anti-GFP IHC. Right**:** *hMYC*, *cd79b*, and *lat*, RNA ISH. Boxed regions in 20X marrow and thymus panels are enlarged in the 60× images directly beneath them. Merged *hMYC*+*cd79b* images are also shown. 2× scale bar = 2 mm; 20× bar = 200 µm; 60× bar = 100 µm. Abbreviations as in Fig. 3, and o= ovary, ba= back, sb= swim bladder

Because Ab recognizing zebrafish lymphocyte proteins are not available, we used RNA in situ hybridization (ISH; RNAscope™) to independently test cell identities using probes for *hMYC* and B cell-specific *cd79b* (Fig. 4A–C). *hMYC* labeled pre-B ALL strongly (Fig. 4A; H&E of this animal shown in Fig. 3D), including cells in the thymus and kidney-marrow (Fig. 4B, C, *pre-B1*). These same areas were also *cd79b*-positive, confirming B-lineage. Thymi of *hMYC* control fish were avidly *hMYC*^*+*^, but had few *cd79b*^*+*^ cells (Fig. 4B, D, *hMYC Ctrl*), indicating thymic B cells are sparse unless pre-B ALL is present. Similarly, *hMYC* control marrow had fewer dually *hMYC*^*+*^/*cd79b*^*+*^ cells (Fig. 4C, D), with normal kidney-marrow architecture, including renal tubules. *hMYC* was absent in the thymus of control *lck:eGFP* fish (Fig. 4B, *lck:eGFP*) proving probe specificity, and showed rare *cd79b*^*+*^ B cells, demonstrating few thymic B cells in WT fish.

We examined a different animal with disseminated pre-BALL and localized T-ALL, based on microscopy and IHC findings (see microscopy, IHC, and H&E in Fig. S4A, *pre-B6*), adding a T-cell-specific probe, *lat*, to distinguish pre-B vs. T-ALL. RNA ISH demonstrated cells that were *hMYC*^+^/*cd79b*^+^/*lat*^-^ had completely replaced the marrow and thymic cortex (Fig. 4E), with GFP^hi^ *hMYC*^+^/*cd79b*^-^/*lat*^+^ cells remaining only in an enlarged thymic medulla (i.e., localized T-ALL).

Similar results were seen in a second animal with near-complete thymic ablation by pre-B ALL (Fig. S4C; Fig. 3F, *pre-B2* shows microscopy and IHC of this specimen). Additional RNA ISH for *cxcr4a*, another gene up-regulated in *hMYC* pre-B vs. T-ALL (Fig. S5A), showed identical staining to *cd79b* and opposite staining to *lat* in two different specimens (Fig. S5B-C), demonstrating *hMYC*^+^/*cd79b*^+^/*cxcr4a*^*+*^/*lat*^-^ cells correspond to B-lineage GFP^lo^ cancers. In summary, in vivo fluorescence, cytometric GFP intensity, qRT-PCR, WB, and RNA ISH all prove dim cancers in *hMYC*;*GFP* fish are pre-B ALL with organ distributions similar to human pre-B ALL.

### Zebrafish pre-B ALL remain GFP^lo^/*lck*^lo^ in every tissue and can be allo-transplanted

**Fig. 5:**
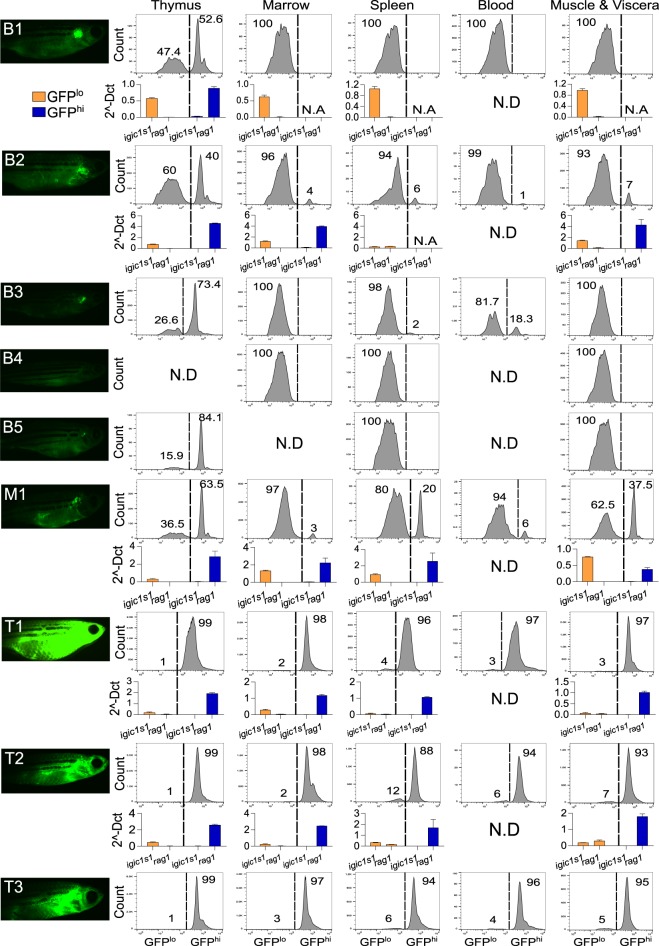
GFP intensity and *igic1s1*/*rag1* distinguish pre-B vs. T-ALL in each anatomic site. Left column shows high-exposure fluorescent microscopy of 4 month-fish with pre-B ALL (B1-5), mixed ALL (M1), or T-ALL (T1-3). Panels at right show flow cytometric analysis of GFP^lo^ and GFP^hi^ cells of thymus, marrow, spleen, peripheral blood, and muscle & abdominal viscera. Each panel shows % of GFP^lo^ vs. GFP^hi^ cells in the entire GFP^+^ gate; 10^5^ events from the lymphoid/precursor gate were analyzed for each plot. N.D.= not determined. Histograms depict expression of *igic1s1* and *rag1* by qRT-PCR in five *hMYC* fish with pre-B ALL (B1, B2), mixed ALL (M1) or T-ALL (T1, T2). Results are normalized to housekeeping genes (*β-actin* and *eef1a1l1*) and shown as mean + Standard Error (S.E.). N.A.= not available due to insufficient cells for RNA extraction

Pre-B ALL disseminated aggressively (Fig. 3D–F, 4, S3A, S4A, C, S5B-C). To test whether pre-B ALL cells retained a GFP^lo^/*lck*^lo^ phenotype in every niche, we examined thymus, marrow, spleen, blood, and muscle and viscera by flow cytometry (Fig. 5). Fish with pre-B ALL (*n* = 5, B1–5) showed GFP^lo^ cells in each tissue, with GFP^hi^ cells (i.e., normal T cells) primarily present only in thymus. As in mammals, zebrafish thymus is the normal site of T cell development, explaining its high content of GFP^hi^ cells. Despite this, some fish (e.g., B1, B2) showed considerable GFP^lo^ populations in thymus, which we consider to be pre-B ALL with thymic invasion. In contrast, fish M1 exhibited a range of GFP^lo^ (37–97%) and GFP^hi^ (3–64%) cells in each tissue, with both populations abundant in non-lymphoid muscle and viscera, where B and T cells are typically scarce. Therefore, we conclude M1 had mixed-ALL, with widespread pre-B ALL, as well as a distinct T-ALL that had spread to muscle and viscera, but not marrow or peripheral blood. In contrast, *hMYC* T-ALL controls (*n* = 3, T1-3) exhibited near-exclusively GFP^hi^ populations in every tissue.

To conclusively test whether GFP^lo^ and GFP^hi^ cells in every anatomic site always represent B-lineage vs. T-lineage cell identities, we analyzed *igic1s1* and *rag1* in FACS-purified GFP^lo^ and GFP^hi^ cells from five of these fish (B1, B2, M1, T1, T2). As previously [Fig. 3A(e), S2A(e)], dim cells from every tissue expressed only *igic1s1*, and only bright cells were *rag1*^+^, including the GFP^lo^ and GFP^hi^ ALLs of M1. Thus, GFP reliably reflects pre-B vs. T-ALL in any niche, establishing *hMYC*;*GFP* zebrafish as a new and novel model to study both ALL types in one genetic context, or even one animal.

To test whether *hMYC* pre-B ALL were truly malignant, we transplanted FACS-purified GFP^lo^ cells into sub-lethally-irradiated immunocompromised wild-type (WT) host fish. Multiple pre-B ALL engrafted (*n* = 9/11), remained GFP^lo^, and continued to express only B-lineage markers (Fig. S6A). As reported for Myc-induced T-ALL [30, 31], we also serially transplanted *hMYC* pre-B ALL, which likewise retained GFP^lo^ B cell identities (Fig. S6A). Interestingly, pre-B ALL exhibited lower leukemia-initiating cell (LIC) frequencies compared to those described for T-ALL [13, 30], although this may relate to the inherent difficulty of detecting engraftment by dimly-fluorescent cancers (Fig. S6B). Overall, these results indicate that GFP^lo^ pre-B ALL are malignant, and that their cellular identities are phenotypically stable.

### MYC-induced zebrafish pre-B and T-ALL have distinct expression signatures

To test whether *D. rerio* and human pre-B ALL share similar gene expression, we next defined GEPs in a new cohort of animals, quantifying 96 transcripts (93 experimental genes plus 3 housekeeping controls) that distinguish B/T/NK cells, lymphoblasts, and precursor populations (genelist in Table S1) [31, 32]. We FACS-purified 8 pre-B ALL, 4 T-ALL, and 2 ALL from a mixed-ALL fish (Fig. S7A–C; GFP^lo^ or GFP^hi^ populations in orange or blue, respectively), as well as control lymphocytes. GFP^hi^ or GFP^lo^ ALL purifications contain mostly malignant cells, but control lymphocytes are less homogeneous; we predicted GFP^hi^ lymphocytes would be “T-cell enriched” and GFP^lo^ cells “B-enriched.” To test the accuracy and reproducibility of GFP-based sorting, we re-analyzed FACS-purified thymic and marrow lymphocytes from WT and *hMYC* fish by flow cytometry, confirming GFP^lo^ and GFP^hi^ cells are highly enriched for these populations (Table S2).

For T cell controls, we isolated GFP^hi^-enriched thymocytes from three 10-fish cohorts of control *hMYC* and *lck*:*GFP* fish (*hMYC* thymus-GFP^hi^, WT thymus-GFP^hi^; Fig. S7D). As another T cell control, we pooled lymphoid-gated [23] GFP^hi^-enriched marrow cells from these same 30 WT fish (WT marrow-GFP^hi^; Fig. S7E, blue). *hMYC* marrow lacked GFP^hi^ cells (Fig. S7F), so these were not analyzed. For B cells, we purified GFP^lo^ marrow cells from the same 3 *hMYC* control cohorts (*hMYC* marrow-GFP^lo^; Fig. S7F) and from marrow of the 30 WT fish used for thymocyte preparations (WT marrow-GFP^lo^; Fig. S7E, orange). In addition, we also analyzed GFP^-^ cells from WT and *hMYC* marrow control samples (WT marrow-GFP^-^; *hMYC* marrow-GFP^-^, Fig. S7E-F), to investigate which cell lineage(s) were enriched in this sub-fraction.

**Fig. 6:**
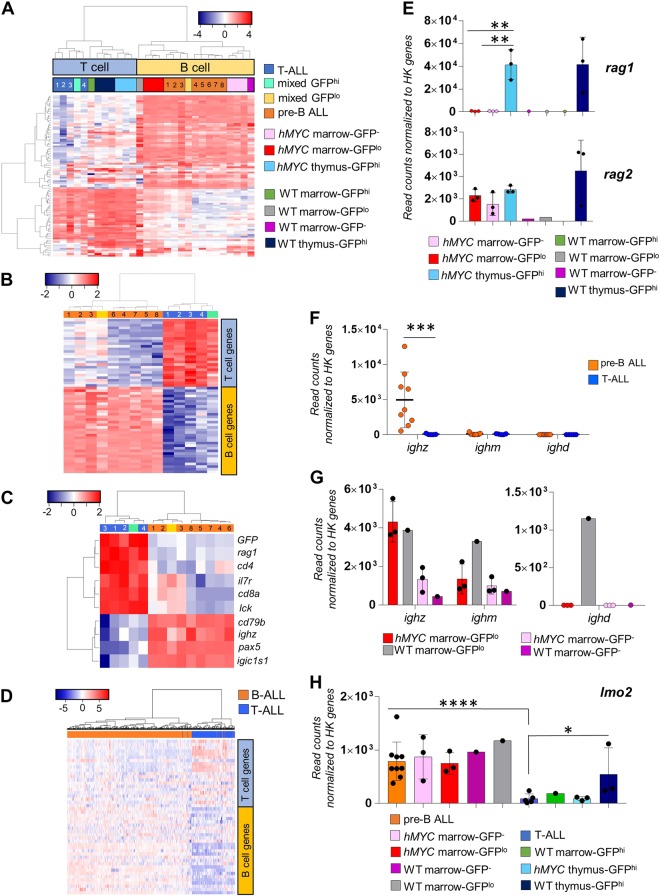
*hMYC* drives pre-B and T-ALL with distinct GEP and alters B-lineage expression. **A** Unsupervised analysis of all malignant and normal lymphocyte populations (*n* = 29) based on GEPs of 93 experimental genes (*β-actin, eef1a1l1, gapdh* housekeeping genes used for normalization not shown). Each sample groups as T- (blue box at top; *n* = 12) or B-lineage (yellow box; *n* = 17). Gene order listed in column D of Table S1. **B** Supervised analysis using significant (FDR<0.05) genes (n=59; order in Table S3) distinguishing pre-B vs. T-ALL. Pre-B (orange; n=8), T- (blue; *n* = 4), and two ALL from a mixed-ALL (GFP^lo^=yellow, GFP^hi^ =green) cluster as pre-B or T-ALL. **C** Analysis with genes from prior qRT-PCR testing (Fig. 3A, S2A). **D** Unsupervised clustering of human ALL from patients on the MILE1 study, using homologues of zebrafish genes that distinguish *hMYC* pre-B vs. T-ALL (human genes in same order as Table S3). **E** *rag1* (top) and *rag2* (bottom) expression in *hMYC* and WT control B and T cell populations. **F** *ighz*, *ighm* and *ighd* levels in pre-B (orange) and T-ALL (blue). **G** *ighz*, *ighm* and *ighd* expression in GFP^lo^ (red) or GFP^-^ (pink) non-malignant *hMYC* B cells, and GFP^lo^ (grey) or GFP^-^ (fuchsia) WT B cells. **H** *lmo2* levels in all samples (*n* = 29) showing highest expression in B-lineage groups and lowest expression in T-ALL. **E**–**H** Mean values are shown ±S.D., after normalization to *β-actin, eef1a1l1*, and *gapdh* housekeeping genes. Significant differences noted (Mann–Whitney test, *p-*values: *<0.05, **<0.01, ***<0.001, ****<0.0001)

Using barcoded gene-specific probes (Nanostring nCounter™), we quantified mRNA levels of 93 transcripts in all 29 samples. Unsupervised analysis clustered all GFP^lo^ and GFP^hi^ triplicate controls tightly (*hMYC* thymus-GFP^hi^, WT thymus-GFP^hi^, *hMYC* marrow-GFP^-^, *hMYC* marrow-GFP^lo^), proving high reproducibility of biologic replicates (Fig. 6A). In addition, each of the 29 samples segregated unambiguously as B-enriched or T-enriched, with every GFP^-^/GFP^lo^ sample (*n* = 17) expressing B-lineage transcripts, and all GFP^hi^ samples (*n* = 12) enriched for T-lineage gene expression. This illustrates that GFP^hi^ control lymphocytes are highly enriched for T cells, while GFP^-^ and GFP^lo^ populations are both enriched for B cells. This was true both for control fish or fish with ALL, irrespective of whether cells were from thymus or marrow, proving the value of *lck:GFP* in this system. Notably, GFP^-^ and GFP^lo^ cells from WT and *hMYC* animals all exhibited similar B-lineage profiles, with *hMYC* GFP^lo^ B cell GEPs most similar to pre-B ALL (Fig. 6A).

Pre-B and T-ALL GEPs were distinct (Fig. 6B; Table S3 lists differentially-expressed genes), with the GFP^hi^ ALL (green) and GFP^lo^ ALL (yellow) of the mixed-ALL animal grouping as T- or pre-B ALL, respectively. In total, ~60 homologous genes able to distinguish human [32] and zebrafish [31] B vs. T cells likewise categorized *hMYC* pre-B vs. T-ALL. Key classifier-genes (Fig. 6C) matched prior qRT-PCR results (Fig. 3A, S2A), with both ALL types showing comparable levels of *rag2*, *hMYC*, and *shmt2*, a known direct MYC target (Fig. S8A) [21], reinforcing that *hMYC* levels and activity are similar in this dual pre-B/T-ALL model. To further examine conservation of gene expression between both types of *D. rerio* and human ALL, we also tested whether human homologues of the differentially-expressed *hMYC* pre-B- vs. T-ALL genes could reliably classify ALL from the MILE1 study (Fig. 6D). This signature exhibited remarkable classification power, separating nearly all of these human ALL correctly. Homologues of several other hematopoietic stem/progenitor- or immature lymphocyte-specific genes showed no significant differences between *hMYC* ALL types (Fig. S8B).

Like ALL, control *hMYC* and WT thymocytes expressed more *rag1* than B cell controls (Fig. 6E, top). From this result, we deduce higher *rag1* in zebrafish T-ALL vs. pre-B ALL is unrelated to malignancy, but a normal feature of *D. rerio* lymphoblasts, although different from mammals.

Notably, *hMYC* B cell controls showed higher *rag2* than WT B cells, implying *hMYC* may expand the pre-B cell population (Fig. 6E, bottom). We also analyzed Ig heavy chain expression.

Surprisingly, pre-B ALL expressed only *ighz* (Fig. 6F), an isotype unique to teleost fish that is functionally analogous to mammalian IgA [33]. GFP^lo^ *hMYC* B cells expressed *ighm* (Fig. 6G, left), so *ighm*^+^ pre-B ALL should be detectable in *hMYC* fish—but these were not found. Therefore, MYC may be oncogenic in only the *ighz*-lineage. Another B-lineage abnormality we noted is that *hMYC* GFP^lo^ cells lacked *ighd* (Fig. 6G, right), so *hMYC* may suppress *ighd* transcription or repress this lineage, just as it seems to block the T cell lineage in *hMYC* marrow (Fig. S7F). Overall, *hMYC* dramatically perturbs zebrafish B cell development, inducing ALL in *ighz*^+^ B cells, and skewing both the *ighm* and *ighd* lineages.

Finally, we found substantially higher *lmo2* in normal and malignant B vs. T cells (Fig. 6H), mirroring *LMO2* findings in human B vs. T cells [34], which we independently confirmed in public human B and T cell data (Fig. S8C). This contradicts the assertion that MYC-driven zebrafish T-ALL emulates the human TAL1/LMO subtype [6]. Instead, *hMYC* T-ALL actually had the lowest *lmo2* levels of the nine populations that were tested, including all T cell controls (Fig. 6H).

## Discussion

Pre-B ALL is the most common pediatric cancer and kills more children than any other type [3], but no good zebrafish models exist for this important disease. Drug screens [17,  18], genetic studies [11, 12, 15] and stem cell discoveries [13, 14] were all made possible by zebrafish T-ALL models, and these and other approaches would likely be similarly fruitful with *D. rerio* pre-B ALL. Here, we describe the first robust zebrafish pre-B ALL model. Unexpectedly, young 3–6 month *hMYC* fish—used for years in several of the aforementioned T-ALL studies—also develop highly-penetrant pre-B ALL.

Remarkably, this went unrecognized for over a decade, raising the possibility that some prior T-ALL studies may have included mixed ALL (see Fig.1). Notably, in lines using the *rag2:GFP* marker instead of *lck:GFP* [6, 10], pre-B and T-ALL would be predicted to have identical fluorescence. Conversely, in other work using *lck:GFP* as a marker [31], unless investigators intentionally purified GFP^lo^ cells, B cells may have been omitted, since B cell GFP is <10% of that in T cells. In fact, even fish with massive pre-B ALL disease burdens exhibited barely-detectable fluorescence (Fig. S3A). Crucially, our data demonstrate that MYC can drive both T-lineage and B-lineage leukemogenesis. This is not surprising, as human B and T cell leukemias all express *MYC* at high levels (*p-*value = 0.34; Fig. S2E). Recent studies likewise demonstrate key roles for MYC in B-lineage ALL molecular pathogenesis [35, 36].

In terms of detecting pre-B ALL, dual-transgenic *hMYC*;*GFP* fish proved particularly valuable to our study, because their *lck:GFP* expression not only allowed pre-B ALL to be detected, but their differing GFP levels also distinguished pre-B and T-ALL in vivo. This dichotomy in *lck* expression extends to normal B and T cells as well, is corroborated by flow cytometry, and corresponds precisely to B-lineage and T-lineage GEPs (Fig. 1E, 2–6, S1, S4). Consequently, even in fish with concomitant pre-B and T-ALL—which we believe are unique to this model—these cell lineages and ALL types, can be reliably separated for independent study.

Apart from the utility of *lck:GFP* in this system, *hMYC* pre-B ALL are powerful because they emulate this human disease in several ways: histology and organ involvement (Figs. 3, 4, S3–5), *lck* levels (Fig. S2B–D), presence of LIC (Fig. S6) and, most importantly, gene expression signature (Fig. 6). In fact, genes that differentiate zebrafish pre-B vs. T-ALL (Fig. 6B) classify human ALL also (Fig. 6D). The GEPs we obtained revealed many transcripts that distinguish pre-B vs. T-ALL in this model, but we also report a two-gene panel to categorize *hMYC* ALL that requires only *igic1s1* and *rag1*, and we note this panel can be applied to any *hMYC* genetic background.

Interestingly, although *RAG1* is expressed by mammalian B-lymphoblasts and T-lymphoblasts, we find that only *D. rerio* T-lymphoblasts express *rag1* highly, with levels >70- and >155-fold greater in normal T or T-ALL cells than in B or pre-B ALL cells, respectively (Fig. 3A(e), S2A(e), 5, 6C,E).

A recent report of zebrafish B cell development despite low *rag1* supports our observation [37]. Apart from *rag1*, pre-B ALL expressed other classic B-lymphoblast genes like *rag2*, surrogate light chain components, *pax5, cd79a/b*, and others (Figs. 3, 4, 6C, S4C, S5B–C, S8A–C). This was true for every dim ALL, including GFP^lo^ mixed-ALL, so we conclude mixed-ALL are co-existing pre-B and T-ALL, and not biphenotypic ALL. Certainly, because *hMYC* can induce both ALL types, it remains possible that mixed-lineage biphenotypic ALL may arise in this system, but to date, our analyses of >40 dim ALL have failed to detect any that express T cell genes, suggesting that GFP^lo^ ALL are always B-lineage, and GFP^hi^ always represent T-ALL. In future work, studying both ALL types in one background—or one animal—presents new opportunities, like efforts to find cooperating genetic lesions unique to one type of ALL, lymphocyte lineage-specific drugs, or myriad other applications.

Our results also demonstrate multiple features of abnormal B and T lymphopoiesis in *hMYC* fish. Of interest, pre-B ALL GEPs closely matched the gene expression pattern of a recently-described *ighz*^+^ B cell population dubbed “fraction 2” [37], suggesting *hMYC* is oncogenically active in this cell population. Supporting this, every pre-B ALL we identified was *ighz*^+^ (Fig. 6F). Liu et al. postulated that the zebrafish *ighm*-lineage lacks a classic pre-B stage. If this is correct, it is logical that pre-B ALL only occurs in *ighz*^+^ cells, the lineage that has pre-B cells. We note that *ighm*^+^ cells do express *lck* (Fig. 6G), so *ighm*^+^ pre-B ALL should be GFP^+^ and detectable in this system. Yet *ighm*^+^ pre-B ALL were never detected, so we conclude they do not occur. In addition to our finding that ALL develops in only *ighz*^+^ cells, we also found that *hMYC* alters other B-lineages, with *ighm* and *ighd* both reduced in *hMYC* marrow (Fig. 6G). Whether this is due to *ighm* and *ighd* transcriptional—or lineage—repression remains to be determined. Notably, T cells are also diminished in *hMYC* marrow (Fig. S7F, Table S2), so *hMYC* alters both the B and T lymphocyte lineages, beyond inducing both pre-B and T-ALL.

Clearly, *hMYC* is leukemogenic to lymphocytes and perturbs zebrafish lymphocyte development. In future work, *hMYC* pre-B ALL can be used for classic zebrafish approaches like chemical and genetic screens, or in mechanistic studies probing *hMYC* biology in either ALL type. *MYC* is arguably the most clinically-relevant oncogene, important in many cancers besides ALL [38], but MYC’s contrasting actions in distinct neoplasias remain largely unexplored. We show *hMYC* fish provide a novel system to address this topic via studies of both human ALL types, using a single model.

## Electronic supplementary material

Supplemental Figures

Table S1

Table S2

Table S3

Supplemental M&M
