# Supplementary material for: Simultaneous B and T cell acute lymphoblastic leukemias in zebrafish driven by transgenic MYC: implications for oncogenesis and lymphopoiesis
Source: Leukemia. 2018 Aug 15;33(2):333–47. doi: 10.1038/s41375-018-0226-6 (PMC6365377; doi:10.1038/s41375-018-0226-6)
Supplement: Supplementary file 1 — Supplemental Figures [file 41375_2018_226_MOESM1_ESM.pdf]

Supplementary Materials for

Simultaneous B and T cell acute lymphoblastic leukemias in zebrafish driven by transgenic MYC: implications for oncogenesis and lymphopoiesis

Chiara Borga[1], Gilseung Park[1]†, Clay Foster[1]†, Jessica Burroughs-Garcia[1], Matteo Marchesin[1], Rikin Shah[1], Ameera Hasan[1], Syed T. Ahmed[1], Silvia Bresolin[4], Lance Batchelor[1], Teresa Scordino[2], Rodney R. Miles[3], Geertruy te Kronnie[4], James L. Regens[5] , J. Kimble Frazer[1]*

Correspondence to: Kimble-Frazer@ouhsc.edu

This PDF file includes:

Figs. S1-S8

Table S1-S3 captions (spreadsheet submitted as additional files)

Fig. S1

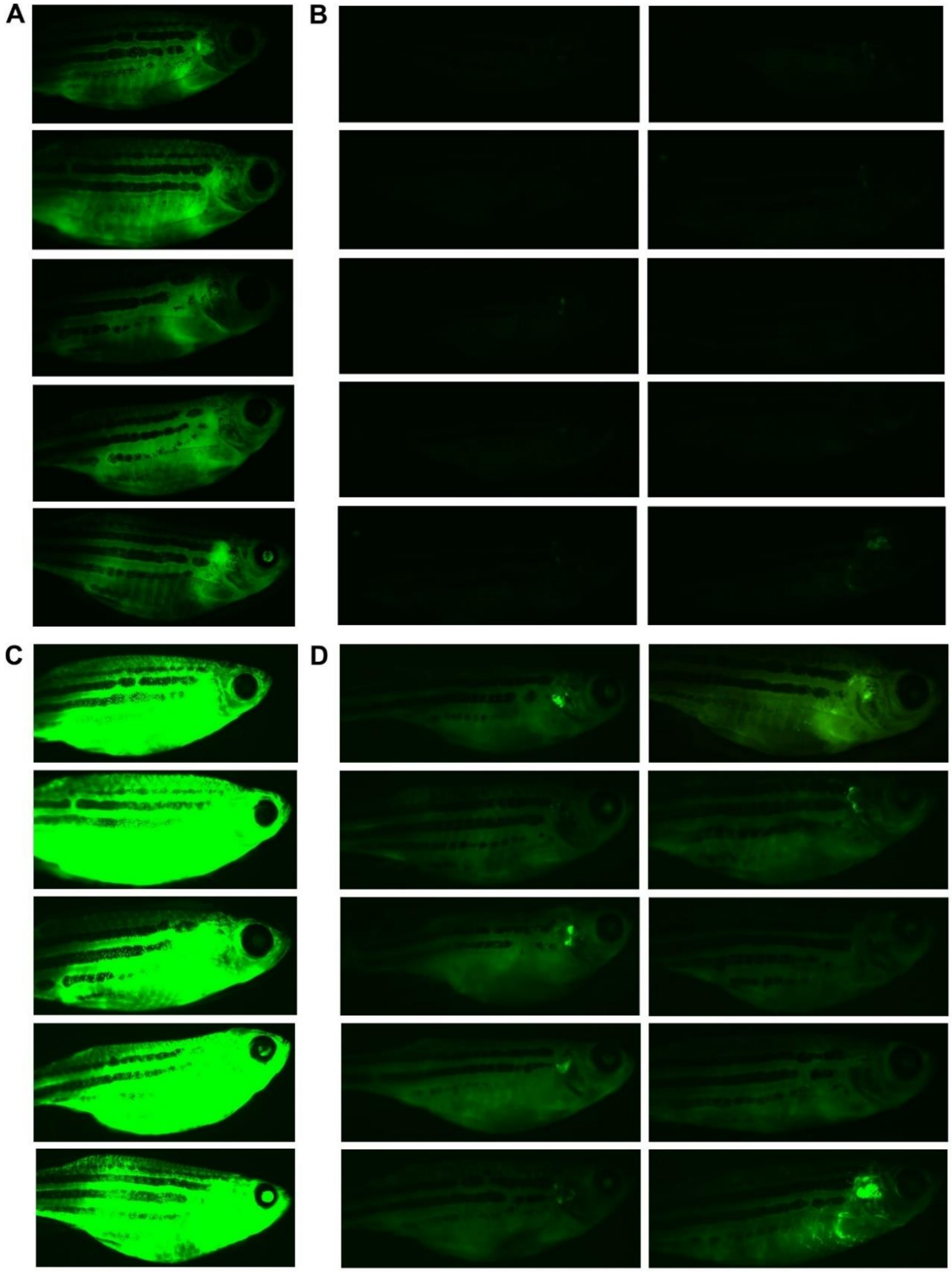

Fig. S1. Fluorescent microscopy images of two types of *hMYC* ALL.

Images of 5 *hMYC* T-ALL using **(A)** low-exposure or **(C)** high-exposure settings and **(B)** low-exposure or **(D)** high-exposure images of 10 *hMYC* pre-B ALL.

Fig. S2

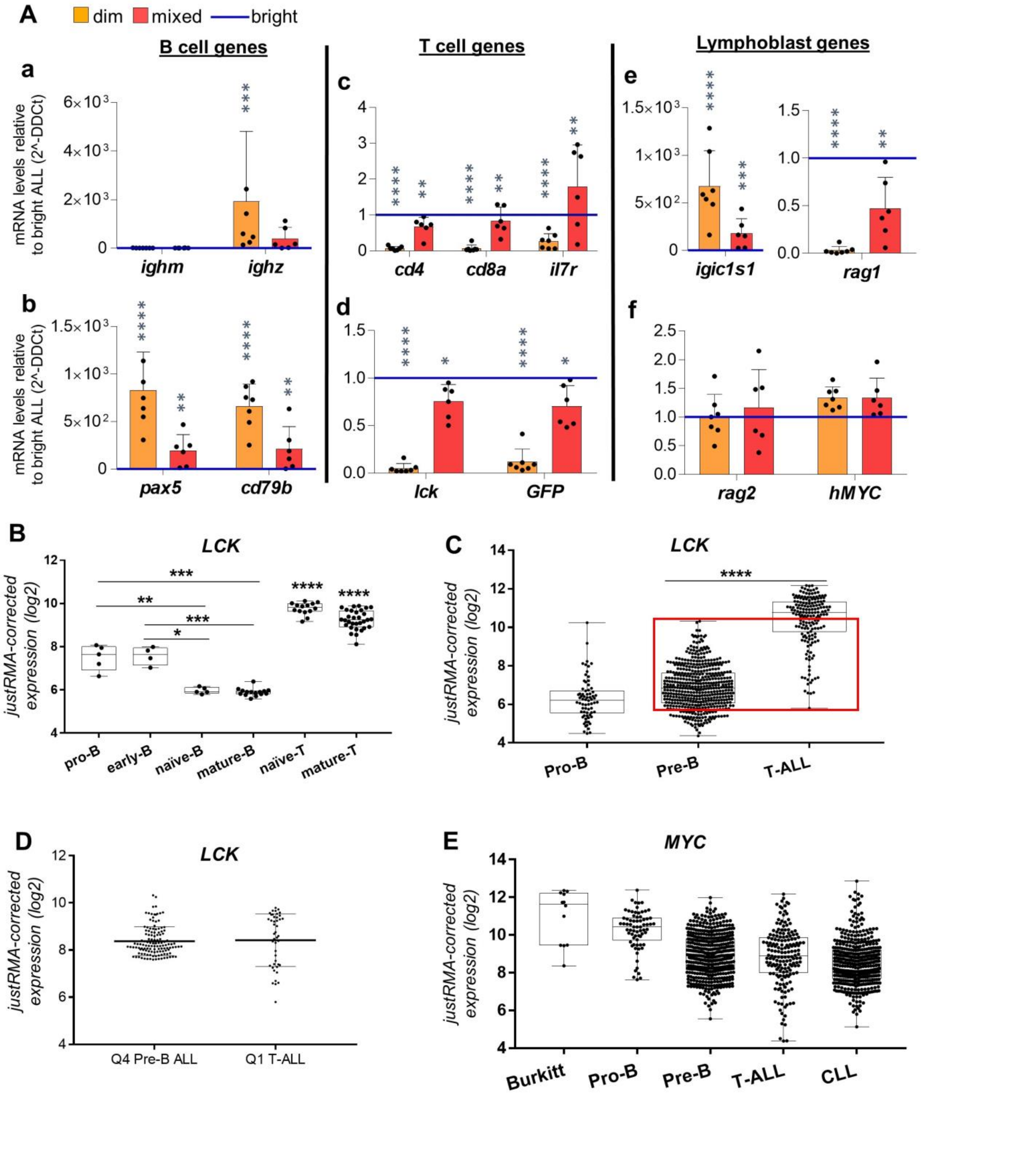
A
dim
mixed
bright
B cell genes
T cell genes
Lymphoblast genes
a
b
c
d
e
f
mRNA levels relative to bright ALL (2^-DDCt)
ighm
ighz
pax5
cd79b
cd4
cd8a
il7r
lck
GFP
igic1s1
rag1
rag2
hMYC
B
LCK
justRMA-corrected expression (log2)
pro-B
early-B
naïve-B
mature-B
naïve-T
mature-T
C
LCK
Pro-B
Pre-B
T-ALL
D
LCK
Q4 Pre-B ALL
Q1 T-ALL
E
MYC
Burkitt
Pro-B
Pre-B
T-ALL
CLL

Fig. S2. Gene expression in zebrafish *hMYC* ALL and *LCK* and *MYC* expression in human normal lymphocytes and lymphoid leukemias.

(**A**) qRT-PCR results of Fig. 3A, depicting gene expression in dim and mixed ALL relative to bright ALL (blue lines). (**B**) *LCK* expression in normal human lymphocytes at different maturation stages. (**C**) *LCK* expression in different human ALL types. Red box highlights pre-B and T-ALL with over-lapping *LCK* expression values. (**D**) Comparison of the highest *LCK*–expressing pre-B ALL quartile (Q4) vs. the lowest T-ALL quartile (Q1) from the MILE1 study shows no statistical difference (*p*-value = 0.75). (**E**) *MYC* expression in different human lymphoid leukemias of the MILE1 cohort. No statistical difference (*p*-value = 0.34) was seen between pre-B and T-ALL. (**B-E**) Expression values are $\log_2$ scale, normalized against the entire dataset using the justRMA algorithm. Statistical analysis using Mann-Whitney test (*p-values*: *<0.05, **<0.01, ***<0.001, ****<0.0001).

Fig. S3

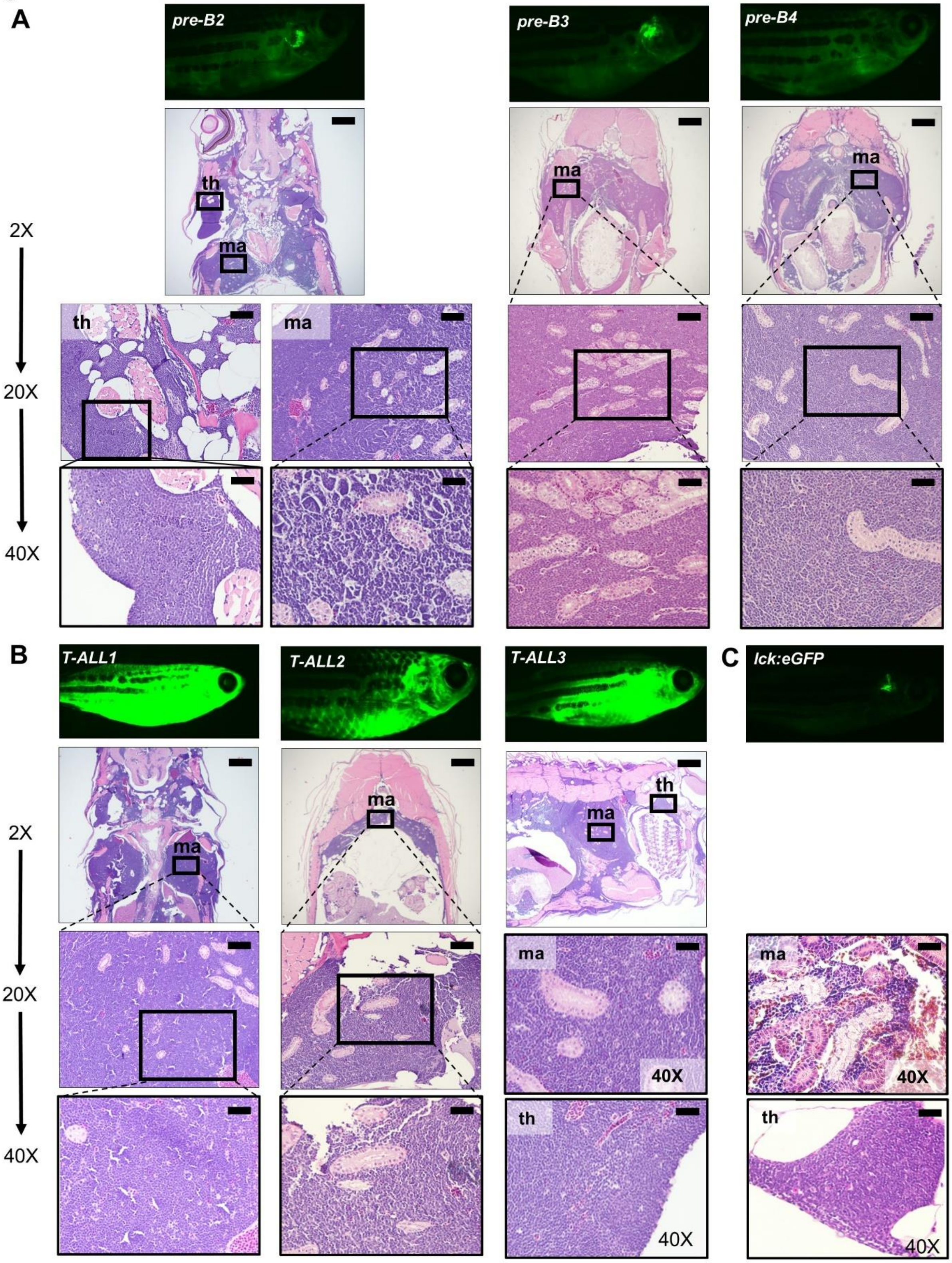
A
pre-B2
pre-B3
pre-B4
th
ma
ma
ma
2X
20X
40X
th
ma
B
T-ALL1
T-ALL2
T-ALL3
C
lck:eGFP
ma
ma
th
ma
2X
20X
40X
ma
40X
ma
40X
th
40X
th
40X

Fig. S3. Histology of *hMYC*-driven pre-B ALL.

High-exposure fluorescent microscopy images and H&E stains of: (**A**) 3 *hMYC* fish with pre-B ALL, (**B**) 3 *hMYC* fish with T-ALL, and (**C**) a WT *lck*:*eGFP* control fish. Vertical series of H&E images in (**A**) and (**B**) have higher magnification moving downward (2X, scale bar = 500 μm; 20X, bar = 50 μm; 40X, bar = 25 μm), except (**B**) *T-ALL3* and (**C**) *lck*:*eGFP* images, where 40X images are shown for both marrow (**ma**) and thymus (**th**). Black boxes in 2X and 20X panels denote regions shown at higher magnification immediately below. Part (**A**) shows 3 pre-B ALL fish (*pre-B2*, sectioned coronally; *pre-B3* and *pre-B4*, sectioned axially). In *pre-B2* 2X image, note effaced architecture and gross enlargement of bilateral thymi and kidney-marrow by basophilic lymphoblasts. In *pre-B3* and *pre-B4* 2X images, marrows are massively expanded by lymphoblast infiltration, displacing skeletal muscle and extending into subcutaneous and cutaneous skin. Part (**B**) shows 3 T-ALL fish (*T-ALL1*, sectioned coronally; *T-ALL2*, sectioned axially; *T-ALL3*, sectioned sagittally) with similar histology to pre-B ALL fish. Part (**C**) shows normal anatomy of kidney-marrow and thymi in WT *lck*:*eGFP* control fish.

Fig. S4

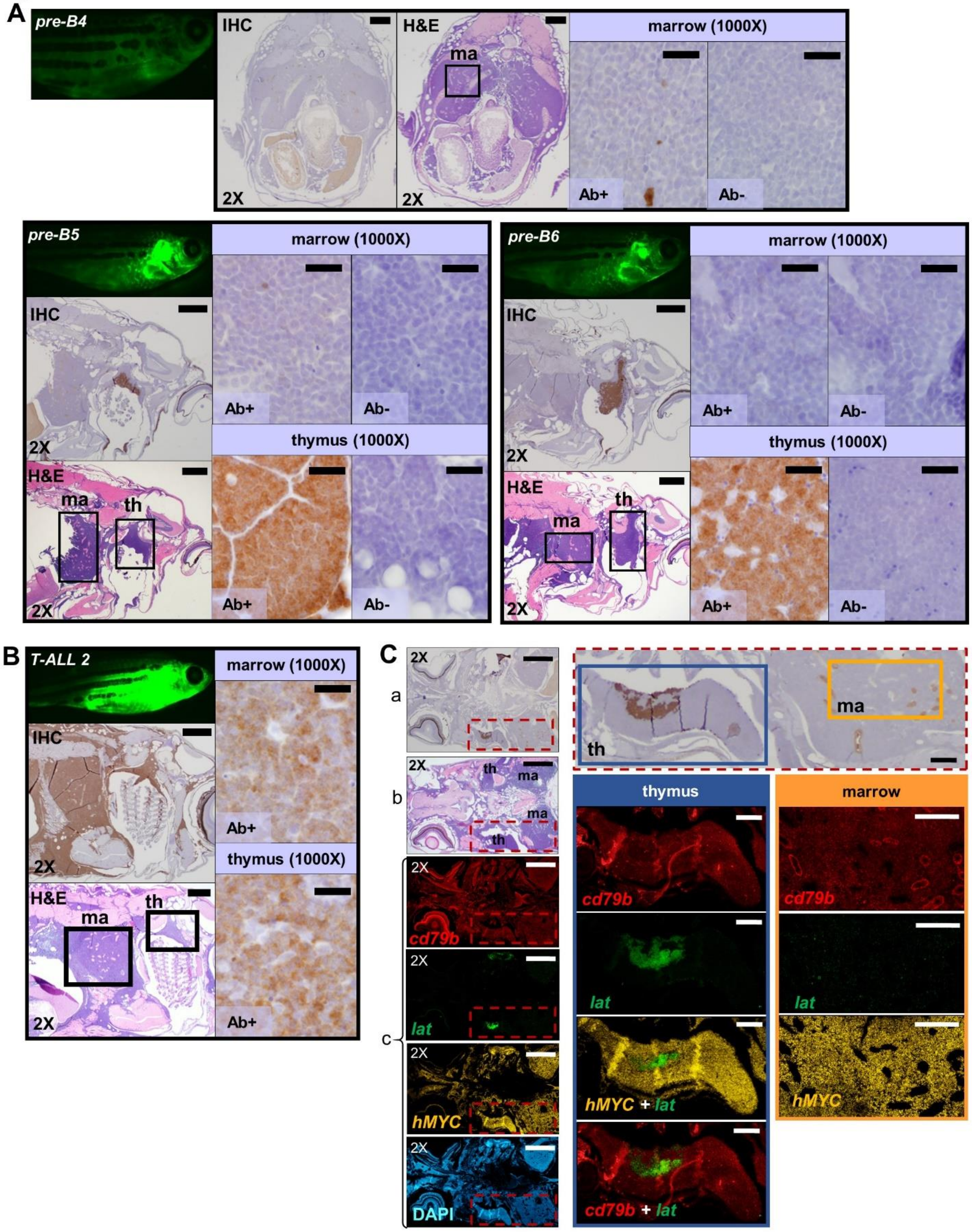
A
pre-B4
IHC
2X
H&E
ma
2X
marrow (1000X)
Ab+
Ab-
pre-B5
IHC
2X
H&E
ma
th
2X
marrow (1000X)
Ab+
Ab-
thymus (1000X)
Ab+
Ab-
pre-B6
IHC
2X
H&E
ma
th
2X
marrow (1000X)
Ab+
Ab-
thymus (1000X)
Ab+
Ab-
B
T-ALL 2
IHC
2X
H&E
ma
th
2X
marrow (1000X)
Ab+
thymus (1000X)
Ab+
C
a
2X
b
2X
th
ma
ma
th
c
2X
cd79b
2X
lat
2X
hMYC
2X
DAPI
th
ma
thymus
cd79b
lat
hMYC + lat
cd79b + lat
marrow
cd79b
lat
hMYC

Fig. S4. IHC and RNA ISH of *hMYC* fish with pre-B ALL.

(**A**) and (**B**), High-exposure fluorescent microscopy images are shown at upper left, with low power anti-GFP IHC and H&E stains adjacent. Boxed regions in H&E images denote areas shown in high power IHC images of marrow (**ma**) or thymus (**th**) at right. Faint anti-GFP signal in pre-B ALL fish marrow is apparent by comparing IHC with or without anti-GFP primary Ab (Ab+, Ab-; all were treated with secondary Ab). (**A**) Two pre-B ALL fish (*pre-B5*, *pre-B6*) have strong thymic fluorescence and IHC signals. In these animals, IHC signals are seen in only thymus, not marrow. In *pre-B4,* individual GFP^+ T cells can be seen in the pre-B ALL tumor mass (*pre-B4;* H&E of this fish is also shown in Fig. S3A). (**B**) *T-ALL2* shows strong IHC signal in both thymus and marrow. 2X images, scale bar = 500 μm; 1000X images, scale bar = 20 μm. **(C)** IHC, H&E and RNA ISH stains in a coronally-sectioned *hMYC* pre-B ALL fish**.** Left column shows 2X images of: (a) anti-GFP IHC, showing a small cluster of GFP^+ T cells in thymus invaded by pre-B ALL cells, (b) H&E showing pre-B ALL infiltration in both thymi (**th**) and marrow (**ma**), (c) RNA ISH for *cd79b* (Cy5, red; 1st panel), *lat* (fluorescein, green; 2nd panel), *hMYC* (Cy3, yellow; 3rd panel) and DAPI (bottom panel). Panels at right show high magnification IHC and ISH stains of area in red-dashed box of 2X images that includes thymus (blue box) and marrow (yellow box). Staining by the T cell-specific *lat* probe corresponds precisely to the thymic region that was also positive by IHC. 2X images, scale bar = 1 mm; high power images, scale bar = 200 μm. Fig. 3F shows fluorescent microscopy and additional IHC stains of this specimen (*pre-B2*).

Fig. S5

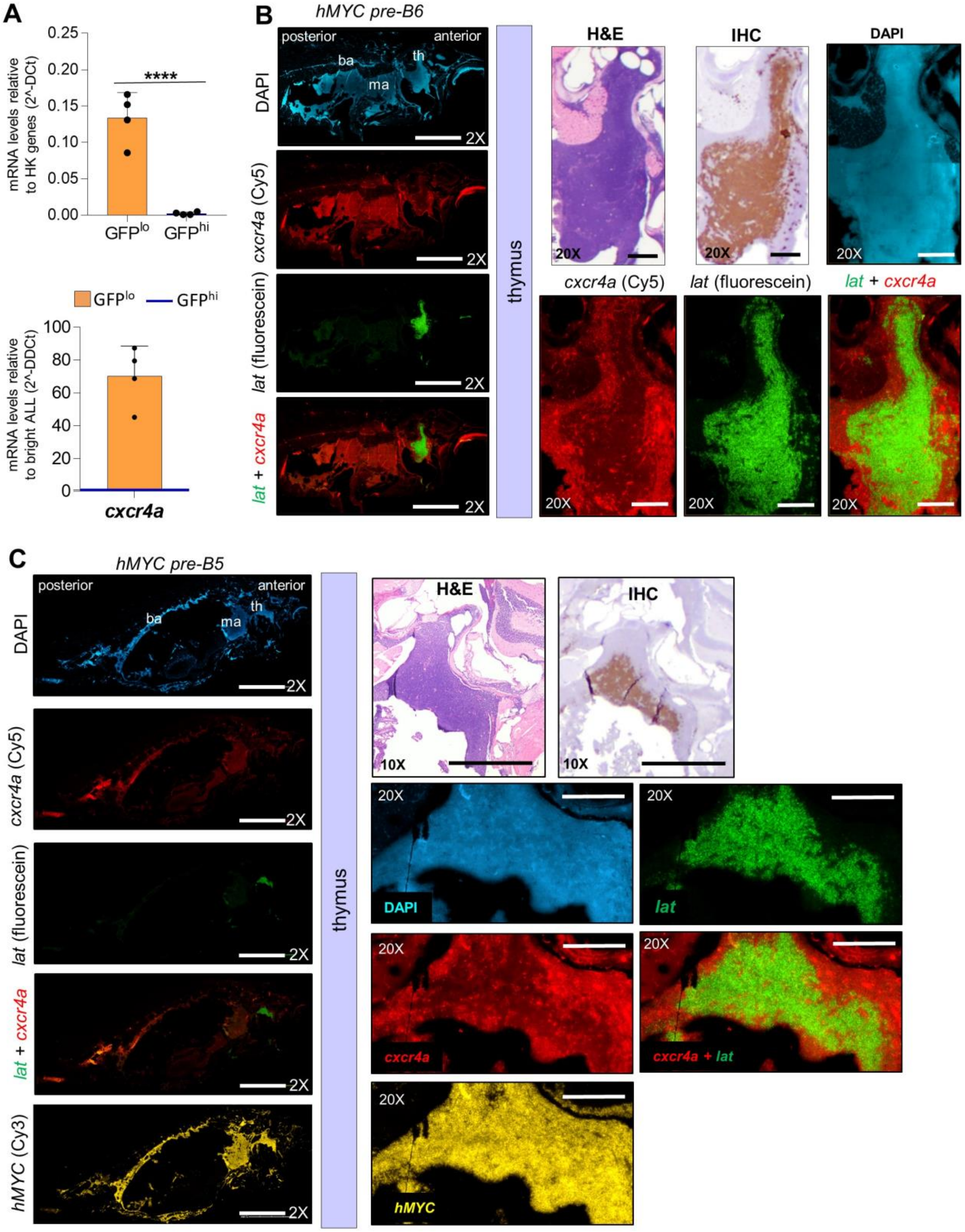
A
0.25
0.20
0.15
0.10
0.05
0.00
mRNA levels relative to HK genes (2^-DCt)
****
GFPlo
GFPhi
100
80
60
40
20
0
mRNA levels relative to bright ALL (2^-DDCt)
cxcr4a
B
hMYC pre-B6
posterior
anterior
ba
th
ma
DAPI
cxcr4a (Cy5)
lat (fluorescein)
lat + cxcr4a
2X
thymus
H&E
IHC
DAPI
20X
cxcr4a (Cy5)
lat (fluorescein)
lat + cxcr4a
C
hMYC pre-B5
posterior
anterior
ba
ma
th
DAPI
cxcr4a (Cy5)
lat (fluorescein)
lat + cxcr4a
hMYC (Cy3)
2X
thymus
H&E
IHC
10X
20X
DAPI
lat
cxcr4a
cxcr4a + lat
hMYC

Fig. S5. Pre-B ALL co-express *hMYC* and B cell-specific *cxcr4a*.

(**A**) qRT-PCR shows significant up-regulation of *cxcr4a* in pre-B (GFP^{lo}) vs. T-ALL (GFP^{hi}) ALL; *cxcr4a* expression is depicted after normalization to housekeeping (*β-actin* and *eef1a1l1*) genes (2^-DCt, top graph) and relative to T-ALL (2^-DDCt, bottom graph). Significant differences are indicated (Mann-Whitney test, *p*****<0.0001). (**B-C**) H&E, anti-GFP IHC, and RNA ISH of two sagittally-sectioned *hMYC* fish with pre-B ALL**. B-C, Left:** total body DAPI stains (top) and RNA ISH for *cxcr4a* (red), *lat* (green), merged *cxcr4a* + *lat* and *hMYC* [yellow; panel C only (*hMYC stain* of specimen *pre-B6* shown in Fig. 4E). **B-C, Right:** 20X images of thymus by H&E, anti-GFP IHC, and RNA ISH of *cxcr4a*, *lat*, and *hMYC* (panel C only). Compare *cxcr4a* results with *cd79b* RNA ISH in Figs. 4E and S4C. (**B**) Specimen *pre-B6* (fluorescent microscopy, additional IHC, and H&E in Fig. S4A; RNA ISH of *cd79b* in Fig. 4E). (**C**) Specimen *pre-B5* (fluorescent microscopy, IHC, and H&E in Fig. S4A). 2X scale bar = 2 mm; 10X = 500 µm; 20X = 200 µm. Abbreviations: **th** = thymus; **ba** = back; **ma** = marrow.

Fig. S6

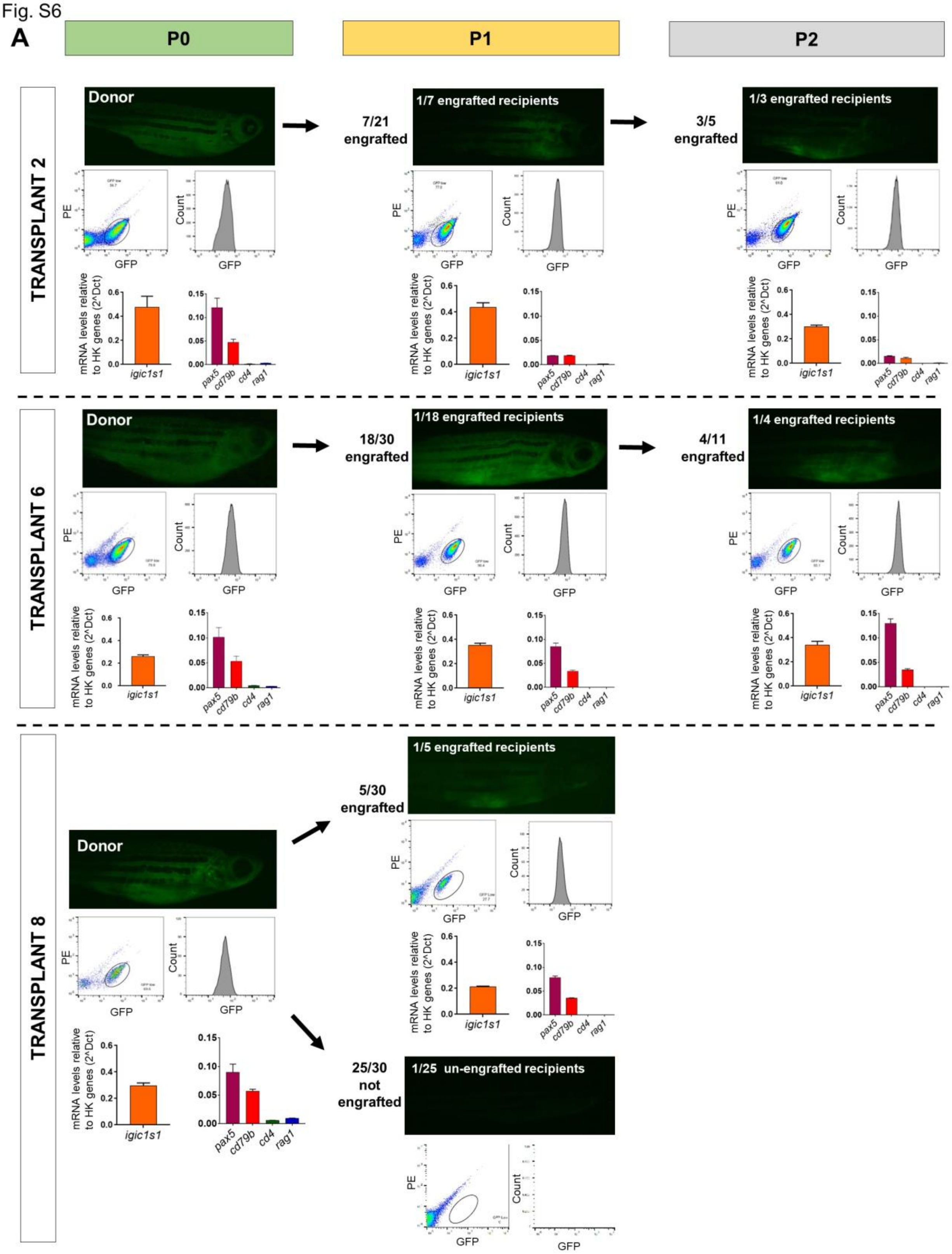
A
P0
P1
P2
TRANSPLANT 2
Donor
7/21 engrafted
1/7 engrafted recipients
3/5 engrafted
1/3 engrafted recipients
TRANSPLANT 6
18/30 engrafted
1/18 engrafted recipients
4/11 engrafted
1/4 engrafted recipients
TRANSPLANT 8
5/30 engrafted
1/5 engrafted recipients
25/30 not engrafted
1/25 un-engrafted recipients
PE
GFP
Count
mRNA levels relative to HK genes (2^Dct)
igic1s1
pax5
cd79b
cd4
rag1

B

Transplant	10K # engrafted/ # injected	25K # engrafted/ # injected	50K # engrafted/ # injected	100K # engrafted/ # injected	250K # engrafted/ # injected	500K # engrafted/ # injected	Total # engrafted	Total # injected
1	0/3	0/2	0/3	0/3	1/3	ND	1	14
2	0/5	0/2	0/4	3/5	ND	4/5	7	21
5	0/3	0/2	0/1	0/4	ND	1/4	1	14
6	ND	1/5	4/6	4/7	5/6	4/6	18	30
7	ND	0/5	0/6	1/7	0/6	0/6	1	30
8	ND	1/5	0/6	1/7	2/6	1/6	5	30

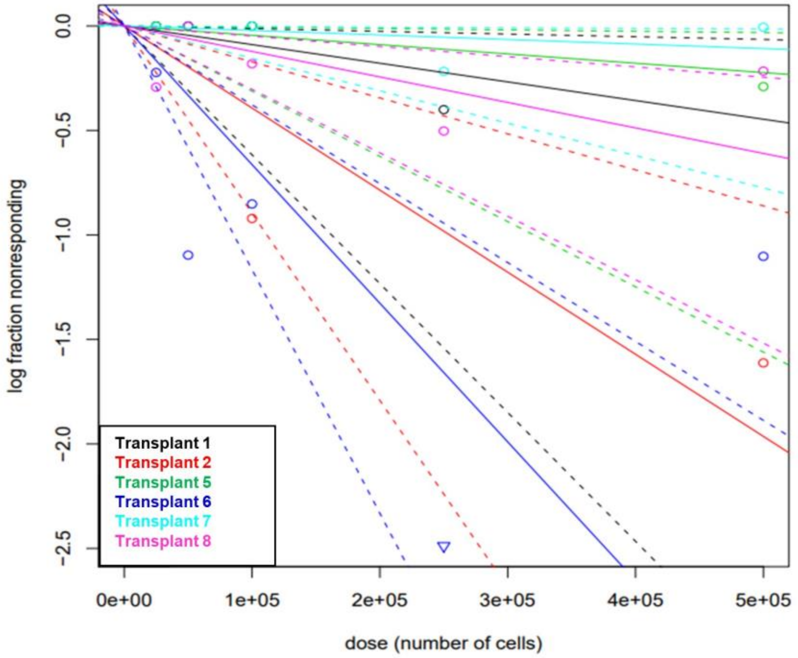

Transplant Series	LIC Frequency	95% CI
1	1:1.1 X 10^6	1:7.7 X 10^6-1:1.6X 10^5
2	1:2.5 X 10^5	1:5.8 X 10^5-1:1.1 X 10^5
5	1:2.2 x 10^6	1:1.5 X 10^7-1:3.2 X10^5
6	1:1.5 X 10^5	1:2.6 X 10^5-1:8.5 X10^4
7	1:4.6 X 10^6	1:3.3 X 10^7-1:6.4 X10^5
8	1:8.2 X 10^5	1:2.0 X 10^6-1:3.2 X10^5

Fig. S6. Pre-B ALL can be allo-transplanted and contain Leukemic-Initiating Cells (LIC).

(**A**) Select results from 3 (Transplants 2, 6, and 8) of 9 *hMYC* pre-B ALL that successfully engrafted after transplant into immunocompromised WT fish (11 pre-B ALL were tested: 9 engrafted and 2 did not engraft). High exposure fluorescent microscopy images, FACS plots showing gating (black ovals) and GFP intensities of sorted populations, and qRT-PCR of B cell- (*igic1/s1*, *pax5*, *cd79b*) and T cell- (*cd4*, *rag1*) specific genes. Donor fish (P0; left column) and one representative recipient fish from the first transplant round (P1; center column) are shown. For Transplant 8, a representative non-engrafted fish is also shown at bottom. For Transplants 2 and 6, data from second-round serial transplants (P2; right column) are also reported, proving pre-B ALL can serially engraft (3 total serial transplants attempted, all successfully engrafted). qRT-PCR results are normalized to HK genes (*β-actin* and *eef1a1l1*) and shown as means + S.E. (**B**) Upper table depicts engraftment results from 6 pre-BALL transplanted using different cell concentrations to determine LIC (example engrafted fish from Transplants 2, 6, and 8 shown in panel A). No engraftment occurred in Transplants 3 and 4 (data not shown). Figure and lower table show estimated LIC frequencies calculated from the 6 *hMYC* pre-B ALL in the upper table (solid lines). LIC frequencies are shown with 95% confidence intervals (dashed lines).

Fig. S7

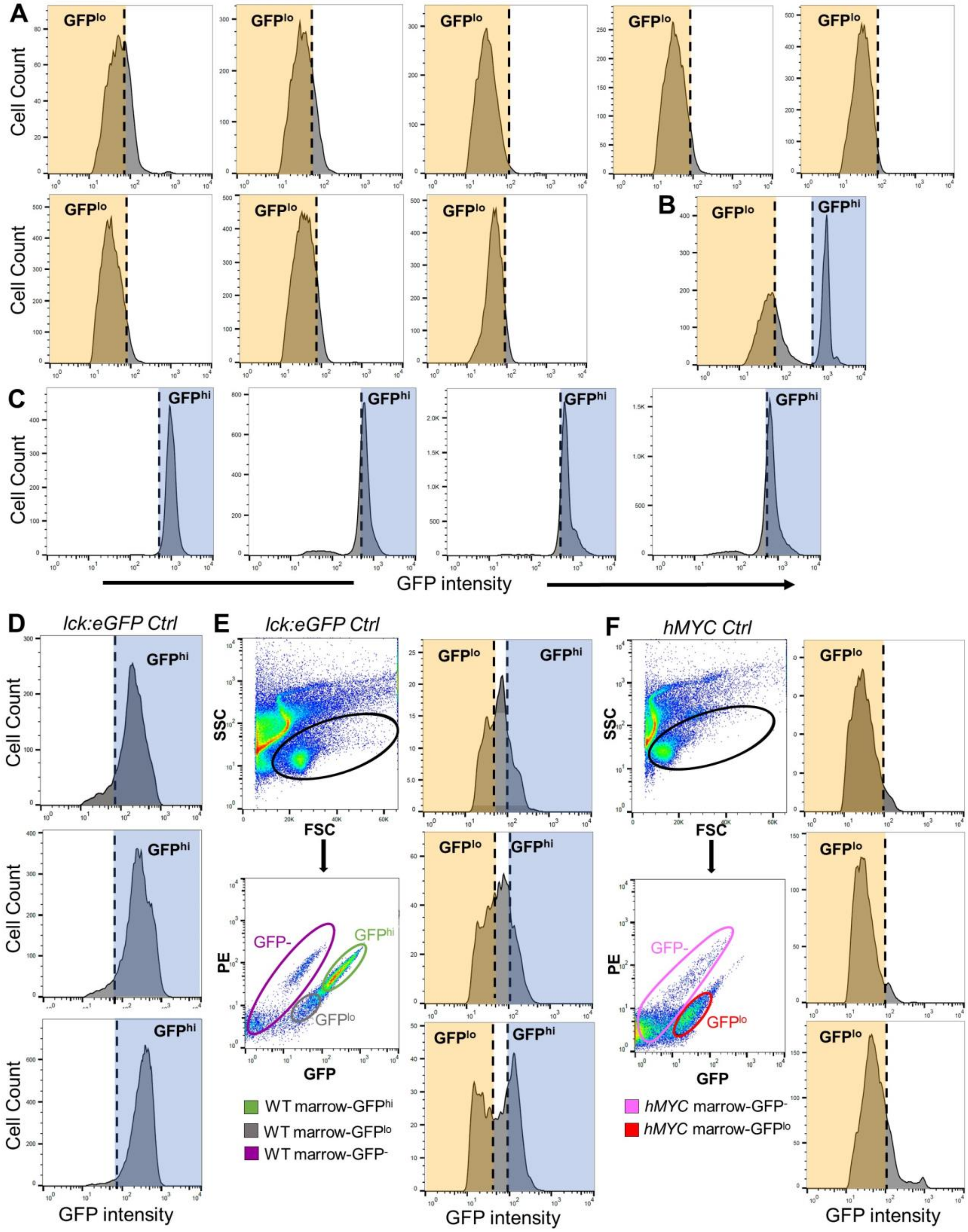
A
B
C
D
E
F
Cell Count
GFPlo
GFPhi
GFP intensity
lck:eGFP Ctrl
hMYC Ctrl
SSC
FSC
PE
GFP
GFP-
WT marrow-GFPhi
WT marrow-GFPlo
WT marrow-GFP-
hMYC marrow-GFP-
hMYC marrow-GFPlo

Fig. S7. GFP^{lo} and GFP^{hi} lymphocytes isolated for expression profiling.

Plots of GFP^{lo} (orange) and GFP^{hi} (blue) populations FACS-purified for RNA quantification. (**A**) Pre-B ALL (n=8) sorted as pure GFP^{lo} populations (orange); (**B**) Mixed ALL sorted as separate GFP^{lo} (orange) and GFP^{hi} (blue) populations; (**C**) T-ALL (n=4) sorted as pure GFP^{hi} populations (blue). Control lymphocyte populations: (**D**) 3 groups of pooled WT *lck:eGFP* thymi (each group, n=10 fish) sorted for GFP^{hi} thymocytes (blue), (**E-F**) 3 groups of pooled WT or *hMYC* marrow (each group, n=10 fish). Upper left panels show side- and forward-scatter (SSC, FSC) gating of the lymphocyte/precursor population (black ovals). Panels below show subsequent purifications of these cells into GFP^{-} (fuchsia and pink ovals), GFP^{lo} (gray and red ovals) and GFP^{hi} (green oval) sub-fractions. Note paucity of GFP^{hi} cells in *hMYC* marrows, which were not isolated. Oval colors match color-coding in Fig. 6. Triplicate plots at right show GFP^{lo} and GFP^{hi} biologic replicates for each 10-fish marrow sample. Marrow cells from each WT group (GFP^{-}, GFP^{lo}, GFP^{hi}) were pooled from all 30 fish to yield sufficient RNA for expression analysis.

Fig. S8

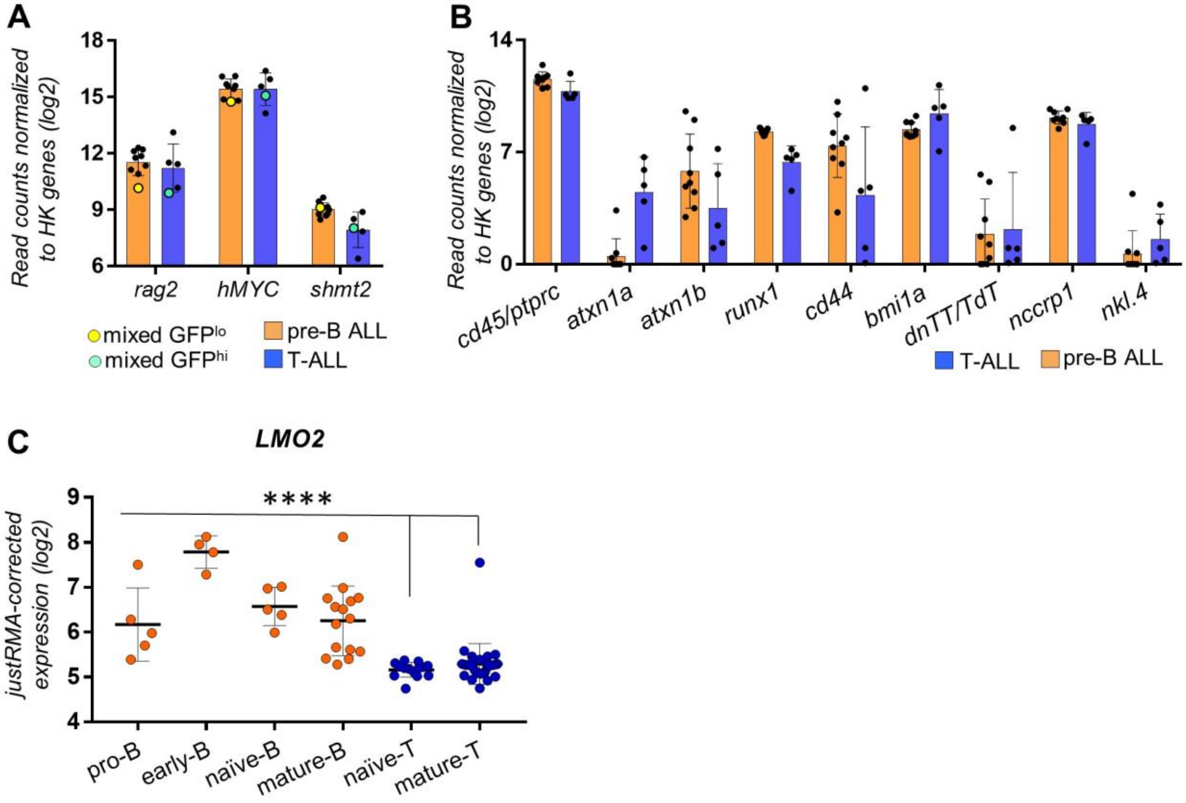

Fig. S8. Gene expression in pre-B ALL and other B and T lymphocytes.

(**A**) Expression of *rag2*, *hMYC* and *shmt2* in pre-B vs. T-ALL. GFP^{lo} and GFP^{hi} ALL from the mixed-ALL are labeled. (**B**) Cell surface marker genes common to B and T cells (*cd45/ptprc, cd44*), genes shared by B- and T-lymphoblasts (*dnTT/TdT*), NK cell genes (*nccrp1*, *nkl.4*), and genes with hematopoietic and/or leukemogenic roles (*atxn1a*, *atxn1b*, *runx1*, *bmi1a*) with similar expression in pre-B and T-ALL. (**C**) Expression of human *LMO2* at different B and T cell stages (Mann-Whitney test, *p*-value: ****<0.0001). Results in A-C expressed as mean ± SD.

Table S1. Expression values of genes tested by Nanostring nCounter RNA hybridization.

Table S2. Purity of FAC-sorted GFP^{hi} and GFP^{lo} populations from Thymus (left) and Marrow (right) of WT and hMYC control fish.

Table S3. Differentially-expressed genes in *hMYC* pre-B ALL and T-ALL.
