# Supplementary material for: Simultaneous B and T cell acute lymphoblastic leukemias in zebrafish driven by transgenic MYC: implications for oncogenesis and lymphopoiesis
Source: Leukemia. 2018 Aug 15;33(2):333–47. doi: 10.1038/s41375-018-0226-6 (PMC6365377; doi:10.1038/s41375-018-0226-6)
Supplement: Supplementary file 5 — Supplemental M&M [file 41375_2018_226_MOESM5_ESM.pdf]

Supplementary Materials for

Simultaneous B and T cell acute lymphoblastic leukemias in zebrafish driven by transgenic MYC: implications for oncogenesis and lymphopoiesis

Chiara Borga[1], Gilseung Park[1]†, Clay Foster[1]†, Jessica Burroughs-Garcia[1], Matteo Marchesin[1], Rikin Shah[1], Ameera Hasan[1], Syed T. Ahmed[1], Silvia Bresolin[4], Lance Batchelor[1], Teresa Scordino[2], Rodney R. Miles[3], Geertruy te Kronnie[4], James L. Regens[5] , J. Kimble Frazer[1]*

Correspondence to: Kimble-Frazer@ouhsc.edu

This PDF file includes:

Supplementary *Material & Methods*

References

Supplementary Material & Methods

Zebrafish Care and Fluorescent Microscopy Screening

Zebrafish housed in an aquatic colony at 28.5°C on a 14:10 hour light:dark circadian cycle and cared for according to protocols approved by the University of Oklahoma Health Sciences Center IACUC (12-066 and 15-046). For all procedures, fish were anesthetized with 0.02% tricaine methanesulfonate (MS-222). 3-6 month old *hMYC;GFP* fish of both genders were screened for abnormal GFP patterns, using a Nikon AZ100 fluorescent microscope. Low exposure (200 ms, 2.8X gain) and high exposure (1.5s, 3.4X gain) settings were used to obtain images with Nikon DS-Qi1MC camera. Images were processed with Nikon NIS Elements Version 4.13 software.

Fluorescence-Activated Cell Sorting (FACS) and Flow Cytometry Analysis

Cells from whole fish, excluding head regions, or specific organs were dissociated using a pestle and passed twice through 35 μm filters. GFP^+ cells were collected from the lymphoid and precursor gates [1] using a BD-FACSJazz Instrument (Becton Dickinson, San Jose, CA, USA). Depending on the experiment performed, we FAC-sorted GFP^+ (GFP^{hi} + GFP^{lo}) cells (microarrays and qRT-PCR, Figs. 1A-D, 3A, and S2A) or GFP^{lo}, GFP^{hi}, and GFP^- populations (allo-transplants and qRT-PCR, Figs. 5; S5, and S6; Nanostring™, Figs. 6 and S8). GFP intensity defined characteristic peaks on either side of ~10^2 on the GFP intensity axis of this instrument; this value was used to discriminate between GFP^{lo} and GFP^{hi} peaks. For percentage calculations, all GFP^+ cells from 10^5 total events in the lymphoid/precursor gate were evaluated. GFP weighted mean fluorescence intensity (wMFI) was calculated using the following formula: [(MFI of GFP^{lo} population x % of GFP^{lo} cells) + (MFI of GFP^{hi} population x % of GFP^{hi} cells)]. For cell purity assays, GFP^{hi} and GFP^{lo} populations were FACS-purified from 3 month WT and *hMYC* control fish thymi and marrow. For each sample, 5,000 events were re-analyzed to test the enrichment of GFP^{hi} or GFP^{lo}

subfractions by the initial FAC-sorting procedure. Flow cytometric analyses performed using FlowJo software (Ashland, OR, USA).

RNA extraction and quantitative real-time polymerase chain reactions

Total RNA was extracted using Trizol according to manufacturer instructions (Invitrogen, Karlsruhe, Germany). For qRT-PCR, 16 ng of total RNA was reverse transcribed using standard methodology. SYBR-Green qRT-PCR was performed using a CFX96 Touch™ Real-Time PCR Detection System (Biorad, Hercules, California, USA). Each cDNA sample was tested in triplicate. The $2^{-DeltaCt}$ (Delta $\mathrm{Ct} = \mathrm{Ct}_{\text{experimental gene}} - \mathrm{Ct}_{\text{housekeeping gene}}$) method [2] was used to calculate the relative expression of each gene to housekeeping genes (*β-actin* and *eef1a1l1*).

RNA Microarrays

In vitro transcription, hybridization to Zebrafish Genome Arrays (#900487), and biotin-labeling performed according to manufacturer instructions (Affymetrix, Santa Clara, CA, USA). Microarray (.CEL file) data generated using Affymetrix GeneChip Command Console Software, normalized against the entire dataset using the justRMA algorithm, and analyzed using R-Bioconductor (Version 3.4.1). Unsupervised and supervised hierarchical clustering used to group specimens based on Euclidean distance and the Ward method. Differentially-expressed genes identified by shrinkage t-tests [3] with local false discovery rate used to correct *p*-values (lfdr <0.05). Data deposited at NCBI GEO repository GSE109437: https://www.ncbi.nlm.nih.gov/geo/query/acc.cgi?acc=GSE109437; enter security token "sbmduyqinlildcz" where indicated). Public microarray data from normal human B and T cells at various maturational stages [4] and leukemia patients on the MILE stage 1 study [5] used for gene expression analysis.

Histology and Immunohistochemistry (IHC)

H&E stains performed using standard methodology. IHC stains performed using 1:8000 dilutions of anti-GFP antibody (#GTX20290, GeneTex, Irvine, CA, USA) at 37° C using a Ventana BenchMark XT instrument. Staining of GFP^{+} tissues performed using an Inview 3,3'-Diaminobenzidine (DAB) detection kit (Ventana Medical Systems, Tucson, AZ, USA).

Western Blot Analysis

FACS-purified GFP^{lo} pre-B ALL and GFP^{hi} T-ALL cells were homogenized in lysis buffer [(20mM Tris-HCl pH 8.0, 137mM NaCl, 2mM EDTA, 1% NP-40, and 10% Glycerol supplemented with Protease Inhibitor Cocktail (#P8340; Sigma, St. Louis, MI, USA)]. Total protein was resolved on a 4–10% gradient polyacrylamide gel and transferred to a nitrocellulose membrane (both BioRad) using transfer buffer (25mM Tris, 192mM glycine, and 20% methanol). Tris-buffered saline, 5% non-fat dry milk, and 0.05% Tween-20, used for blocking. Blots incubated overnight at 4°C with anti-GFP primary antibody (#sc-9996; Santa Cruz, Dallas, TX, USA), followed by incubation with horseradish peroxidase-conjugated secondary antibody (BD). Immuno-reactive bands detected using ECL 2 western blot substrate (Pierce; Waltham, MA, USA). Parallel incubation with anti-β-actin antibody (#ab8227; Abcam, Cambridge, MA, USA) used as a positive control.

Allo-transplantation assays and Leukemia-Initiating Cells (LIC) calculations

For allo-transplants, FACS-purified GFP^{lo} pre-B ALL cells were pelleted by centrifugation and re-suspended in sorting media [1X RPMI medium (Life Sciences technology), 10% FBS, and 1% penicillin and streptomycin] to achieve stock solutions with concentrations of 10^5 cells/µl. Stock solutions were serially diluted to achieve the desired dose for injections into sub-lethally irradiated (23 Gy) recipient *WIK* WT fish 48-72 hours after irradiation. Transplantations were performed

by injecting 2.5-5µl of cell suspension into the peritoneal cavity with a Hamilton syringe. Recipient fish were scored for engraftment by fluorescent microscopy every 7 days until 21 days post-transplant, or until host fish became moribund. Engraftments detected by fluorescent microscopy were confirmed by dissecting fluorescent tissue from recipients and analyzing cell suspensions by FACS and qRT-PCR, in identical fashion to analyses done on fish with *de novo* ALL. LIC frequencies were calculated using limiting dilution analysis software (http://bioinf.wehi.edu.au/software/elda/), with LIC frequencies reported with 95% confidence intervals.

Statistical analysis

GraphPad Prism 7 software (La Jolla, CA, USA) used to calculate Spearman correlations and non-parametric Mann-Whitney tests for genes tested by qRT-PCR. Two-tailed 95% confidence intervals were used to determine significance, and significant differences are reported as *p-values* with *p** <0.05, ** <0.01, *** <0.001 and **** <0.0001.

References

1. Traver D, Paw BH, Poss KD, Penberthy WT, Lin S, Zon LI. Transplantation and in vivo imaging of multilineage engraftment in zebrafish bloodless mutants. *Nat Immunol* 2003 Dec; **4**(12)**:** 1238-1246.

2. Meyer LH, Eckhoff SM, Queudeville M, Kraus JM, Giordan M, Stursberg J*, et al.* Early relapse in ALL is identified by time to leukemia in NOD/SCID mice and is characterized by a gene signature involving survival pathways. *Cancer Cell* 2011 Feb 15; **19**(2)**:** 206-217.

3. Opgen-Rhein R, Strimmer K. Accurate ranking of differentially expressed genes by a distribution-free shrinkage approach. *Stat Appl Genet Mol Biol* 2007; **6:** Article9.

4. Novershtern N, Subramanian A, Lawton LN, Mak RH, Haining WN, McConkey ME*, et al.* Densely interconnected transcriptional circuits control cell states in human hematopoiesis. *Cell* 2011 Jan 21; **144**(2)**:** 296-309.

5. Haferlach T, Kohlmann A, Wieczorek L, Basso G, Kronnie GT, Bene MC*, et al.* Clinical utility of microarray-based gene expression profiling in the diagnosis and subclassification of leukemia: report from the International Microarray Innovations in Leukemia Study Group. *J Clin Oncol* 2010 May 20; **28**(15)**:** 2529-2537.
